# Polaprezinc combined with clarithromycin-based triple therapy for *Helicobacter pylori*-associated gastritis: A prospective, multicenter, randomized clinical trial

**DOI:** 10.1371/journal.pone.0175625

**Published:** 2017-04-13

**Authors:** Bei Tan, Han-Qing Luo, Hong Xu, Nong-Hua Lv, Rui-Hua Shi, He-Sheng Luo, Jian-Sheng Li, Jian-Lin Ren, Yi-You Zou, Yan-Qing Li, Feng Ji, Jing-Yuan Fang, Jia-Ming Qian

**Affiliations:** 1 Department of Gastroenterology, Peking Union Medical College Hospital, Chinese Academy of Medical Science & Peking Union Medical College, Beijing, China; 2 Department of Gastroenterology, the First Bethune Hospital of Jilin University, Jilin, China; 3 Department of Gastroenterology, the First Affiliated Hospital of Nanchang University, Nanchang, China; 4 Department of Gastroenterology, Jiangsu Province Hospital and First Affiliated Hospital with Nanjing Medical University, Nanjing, China; 5 Department of Gastroenterology, Renmin Hospital of Wuhan University and Hubei General Hospital, Wuhan, China; 6 Department of Gastroenterology, the First Affiliated Hospital of Zhengzhou University, Zhengzhou, China; 7 Department of Gastroenterology, Zhongshan Hospital of Xiamen University, Xiamen, China; 8 Department of Gastroenterology, Xiangya Hospital of Central South University, Changsha, China; 9 Department of Gastroenterology, Qilu Hospital of Shandong University, Jinan, China; 10 Department of Gastroenterology, First Affiliated Hospital of Zhejiang University, Hanzhou, China; 11 Department of Gastroenterology, Renji Hospital Shanghai Jiaotong University School of Medicine, Shanghai, China; University Hospital Llandough, UNITED KINGDOM

## Abstract

The efficacy and safety of polaprezinc combined with triple therapy was compared with triple therapy alone in the eradication of *Helicobacter pylori*. A randomized, parallel-group, open-label, controlled, prospective multicenter study was conducted in 11 cities in China. Treatment-naive patients with *H*. *pylori*–associated gastritis were randomly assigned to one of three arms for a 14-day treatment: Arm A triple therapy (omeprazole 20 mg, amoxicillin 1 g, and clarithromycin 500 mg, each twice daily) plus polaprezinc 75 mg twice daily; Arm B triple therapy plus polaprezinc 150 mg twice daily, or Arm C triple therapy alone. The rate of *H*. *pylori* eradication was the primary endpoint. Secondary endpoints were symptom improvement and lower incidence of adverse events. 303 patients completed the study– 106, 96, and 101 patients in Arms A, B, and C, respectively. Intention-to-treat (ITT) analysis showed that the rate of *H*. *pylori* eradication was significantly higher for Arms A (77.0%) and B (75.9%) compared to Arm C (58.6%) (*P* < 0.01), whereas there was no difference between Arms A and B (*P* = 0.90). Per-protocol (PP) analysis showed that the rate of *H*. *pylori* eradication was significantly higher for Arms A (81.1%) and B (83.3%) compared to Arm C (61.4%) (*P* < 0.01), whereas there was no significant difference between Arms A and B (*P* = 0.62). All three groups reported significant symptom improvement at 7, 14, and 28 days after treatment, compared to baseline (*P* < 0.0001). The adverse event rate for Arm B (5.1%) was higher than for Arms A (2.8%) (*P* = 0.04) and C (1.9%) (*P* = 0.02). There were no serious adverse events in any group. It appears that standard dose polaprezinc combined with triple therapy can significantly improve the *H*. *pylori* eradication rate, without an increase in toxicity.

## Introduction

*Helicobacter pylori (H*. *pylori)*, one of the most globally prevalent pathogens, colonizes about 50% of the world’s population [1]. It is transmitted from human to human and causes chronic gastritis in all colonized subjects. It can lead to peptic ulcer, atrophic gastritis, gastric adenocarcinoma, and MALT lymphoma. *H*. *pylori* eradication cures gastritis and can alter the development of long-term complications [2]. The Kyoto Global Consensus recommends that individuals infected with *H*. *pylori* should be offered eradication therapy unless there are competing considerations [3].

In China, the rate of *H*. *pylori* infection remains elevated at 40%–60%, in part due to increasing rates of antibiotic resistance and decreasing rates of efficacy with proton pump inhibitor (PPI)–based triple therapy regimens [4]. Among the antibiotics recommended for *H*. *pylori* eradication therapy, rates of resistance to metronidazole are as high as 60%–70%, to clarithromycin 20%–38%, and to levofloxacin 30%–38%. Resistance to amoxicillin, furazolidone, and tetracycline remains low at 1%–5%. As rates of antibiotic resistance have increased, the rates of *H*. *pylori* eradication using standard triple therapy (PPI, clarithromycin, and amoxicillin) have declined well below 80%. Extending therapy from 7 to 10 days increases the eradication rate by only about 5%. Common therapies, such as sequential therapy, concomitant therapy, and levofloxacin-based triple therapy have demonstrated no advantage for native Chinese patients [4]. Currently, bismuth-containing quadruple therapy is the recommended front-line treatment in China. However, some patients have an intolerance to bismuth (e.g., allergy) and experience gastrointestinal symptoms such as nausea, vomiting, and darkened stools. Also, the ideal regimen for bismuth (i.e., compound, formulation, dose, dosing interval, and relation to meals) remains unclear [5]. The Fourth Chinese National Consensus Report on the management of *H*. *pylori* infection suggested that combining a mucosal protective agent with triple therapy may have the same efficacy as bismuth-containing quadruple therapy [6]. However, few clinical studies in China have evaluated the efficacy of adding a mucosal protective agent to enhance eradication of *H*. *pylori*.

Polaprezinc is a chelate compound of zinc and l-carnosine, with a history of clinical use in Japan spanning more than 20 years. In addition to its recognized role as a gastric mucosal protective agent that promotes the healing of peptic ulcers, polaprezinc also improves *H*. *pylori* eradication rates [7, 8]. In 1999, Kashimura and colleagues [7] reported that polaprezinc combined with triple therapy comprised of lansoprazole, amoxicillin, and clarithromycin can increase the *H*. *pylori* eradication rate from 24/31 (77.4%, triple therapy alone) to 33/35 (94.3%), with no increase in the incidence of adverse events [7]. Based on this single-center, small sample size study in Japan, we designed and conducted a randomized, parallel-group, controlled, open-label prospective multicenter study to evaluate the clinical efficacy and safety of adding polaprezinc to triple therapy to improve the rate of *H*. *pylori* eradication in Chinese patients with gastritis. We also compared the clinical efficacy of two different doses of polaprezinc combined with triple therapy in the eradication of *H*. *pylori* and improvement of clinical symptoms.

## Materials and methods

### Study design

This was a randomized, parallel-controlled, open-label, prospective multicenter clinical study. The study protocol and the CONSORT checklist are available as supporting documents; see S1–S3 Files. The study adhered to the guidelines of the Declaration of Helsinki. Written informed consent was obtained from all participants prior to enrollment. This study protocol was approved by the Peking Union Medical College Hospital Ethics Committee (HS2013002) (S4 and S5 Files). The study has been retrospectively registered with the Chinese Clinical Trial Registry (ChiCTR-IIR-16009688). Reason for not registering prior to enrollment of patients was due to the fact that it was not a mandatory requirement to have this study registered by our approving Ethics Committee. The authors confirm that all ongoing and related clinical trials for this intervention will be registered prospectively.

### Participants

We prospectively enrolled patients infected with *H*. *pylori* who visited one of the 11 recruited geographically diverse hospitals between January 2014 and June 2015: Peking Union Medical College Hospital, Chinese Academy of Medical Science & Peking Union Medical College; First Clinical Hospital of Jilin University; First Affiliated Hospital of Nanchang University; Jiangsu People’s Hospital; People’s Hospital of Wuhan University; First Affiliated Hospital of Zhengzhou University; Zhongshan Hospital of Xiamen University; Xiangya Hospital of Central South University; Qilu Hospital of Shandong University; First Affiliated Hospital of Zhejiang University; Renji Hospital Shanghai Jiaotong University School of Medicine.

Patients with upper gastrointestinal symptoms who had been referred for a ^13^C or ^14^C urea breath test and esophagogastroduodenoscopy (EGD) were recruited into the study. Patients aged 18–70 years with documented *H*. *pylori* infection and without prior eradication therapy were eligible for enrollment.

Patients who met one or more of the following criteria were excluded: prior standard eradication therapy for *H*. *pylori*; EGD-confirmed gastric or duodenal ulcer, gastric cancer, lymphoma, and obvious erosive or hemorrhagic gastritis within 6 months; prior antibiotic or bismuth therapy within 4 weeks of enrollment; history of gastrostomy; prior PPI therapy within 2 weeks of enrollment; penicillin skin test positive; contraindications or allergy to any of the study drugs; severe liver, heart, or kidney disease, alcoholism, malignancy, or other serious diseases; psychosis, severe neurosis; pregnancy or lactation; participation in other clinical trials within 3 months of enrollment.

### Intervention

All enrolled participants underwent a physical examination and laboratory studies as specified by the protocol, including a complete blood count (CBC), comprehensive metabolic panel, electrocardiogram (ECG), and for women of childbearing age, a urine pregnancy test. Participants were then randomly assigned to Arm A, B, or C. Arm A: polaprezinc 75 mg + omeprazole 20 mg + amoxicillin 1 g + clarithromycin 0.5 g orally twice daily for 14 days; Arm B: polaprezinc 150 mg + omeprazole 20 mg + amoxicillin 1 g + clarithromycin 0.5 g orally twice daily for 14 days; Arm C: omeprazole 20 mg + amoxicillin 1 g + clarithromycin 0.5 g orally twice daily for 14 days. All participants repeated the ^13^C or ^14^C urea breath test, physical examination, and laboratory studies 4 weeks after therapy.

Study drugs were polaprezinc (Ruilaisheng, Broadwell Pharmaceutical, Jilin, China), omeprazole (Losec^®^, AstraZeneca Pharmaceutical, Shanghai, China), amoxicillin (Federal Amoxil, Zhuhai Federal Pharmaceutical, Zhuhai, China), and clarithromycin (Limaixian, Xi’an Lijun Pharmaceutical, Xi’an, China).

### Outcomes

The primary endpoint of this study was *H*. *pylori* eradication 4 weeks after the end of intervention, as measured by a ^13^C or ^14^C urea breath test. The secondary endpoints were the improvement of gastrointestinal symptoms by day 7, 14, and 28 after the completion of treatment and lower incidence of adverse events. Each symptom, including upper abdominal pain, acid reflux, belching, heartburn, bloating, nausea and vomiting, was graded according to its severity: 0 none, 1 mild, 2 moderate, or 3 severe. Participants were asked to assign and record the severity score of each symptom before treatment and on day 7, 14, and 28 after treatment. The participants were also informed of the common adverse events before treatment and were asked to record these during treatment. There were follow-up interviews on day 7, 14, and 28 after treatment. The physical examination and laboratory studies were repeated on day 28 after therapy, including CBC, urine test, comprehensive metabolic panel, and ECG.

### Sample size

The calculation of the sample size was based on the primary endpoint. A previous single-center, small sample size study conducted in Japan determined that polaprezinc combined with triple therapy increased the *H*. *pylori* eradication rate from 77.4% to 94.3% [7]. Using the χ^2^ test, with a two-sided α value of 0.05 and β value of 0.20, we estimated that a sample size of 112 participants in each group would detect a 15% difference in eradication rates.

### Randomization and blinding

When an eligible participant was ready to be randomized, the investigator at each recruited site provided the participant’s name and study ID to an independent researcher. Participants were randomly assigned 1:1:1 to Arm A, B, or C according to the unique randomization schedule that was generated by Department of Epidemiology and Health Statistics, Peking Union Medical College and supplied to each center. The independent researcher was in charge of drug distribution and recovery. This trial was designed as open-labled, for the primary endpoint eradication rate of *H*.*pylori* is objective data.

### Statistical analyses

All statistical analyses were performed by the Department of Epidemiology and Health Statistics, Peking Union Medical College using SAS 9.3 software (SAS Institute, Irvine, CA). The primary endpoint *H*. *pylori* eradication rate and one of the secondary endpoints improvement of gastrointestinal symptoms were analyzed in an intention-to-treat (ITT) population and a per-protocol (PP) population. The secondary endpoint the incidence of adverse events was analyzed with a safety analysis (SAS) population. The ITT population included all participants who were enrolled, randomized, and who took at least one dose of drug. Missing observations were accounted for using the last observation carried forward (LOCF) method. The PP population included all participants who completed the entire protocol with good compliance. The SAS population included all participants who were randomized, took drugs, and were assessed for safety at least once. Categorical variables were described as percentages, while continuous variables were described as means and standard deviations. For the continuous variables that meet and do not meet the parameter test, analysis of variance (ANOVA) and the Kruskal-Wallis test were used, respectively. For the categorical variables that meet and do not meet the χ^2^ test, the χ^2^ test and Fisher exact test were used respectively. The Cochran–Mantel–Haenszel (CMH)–χ^2^ test was used for group comparisons, and the Mantel–Heanszel method was used to calculate the 95% confidence interval (CI) of the *H*. *pylori* eradication rate in each group, and the difference in eradication rates between two groups. All *P* values were two-tailed and *P* < 0.05 was considered statistically significant.

## Results

### Demographics and characteristics

**Fig 1:**
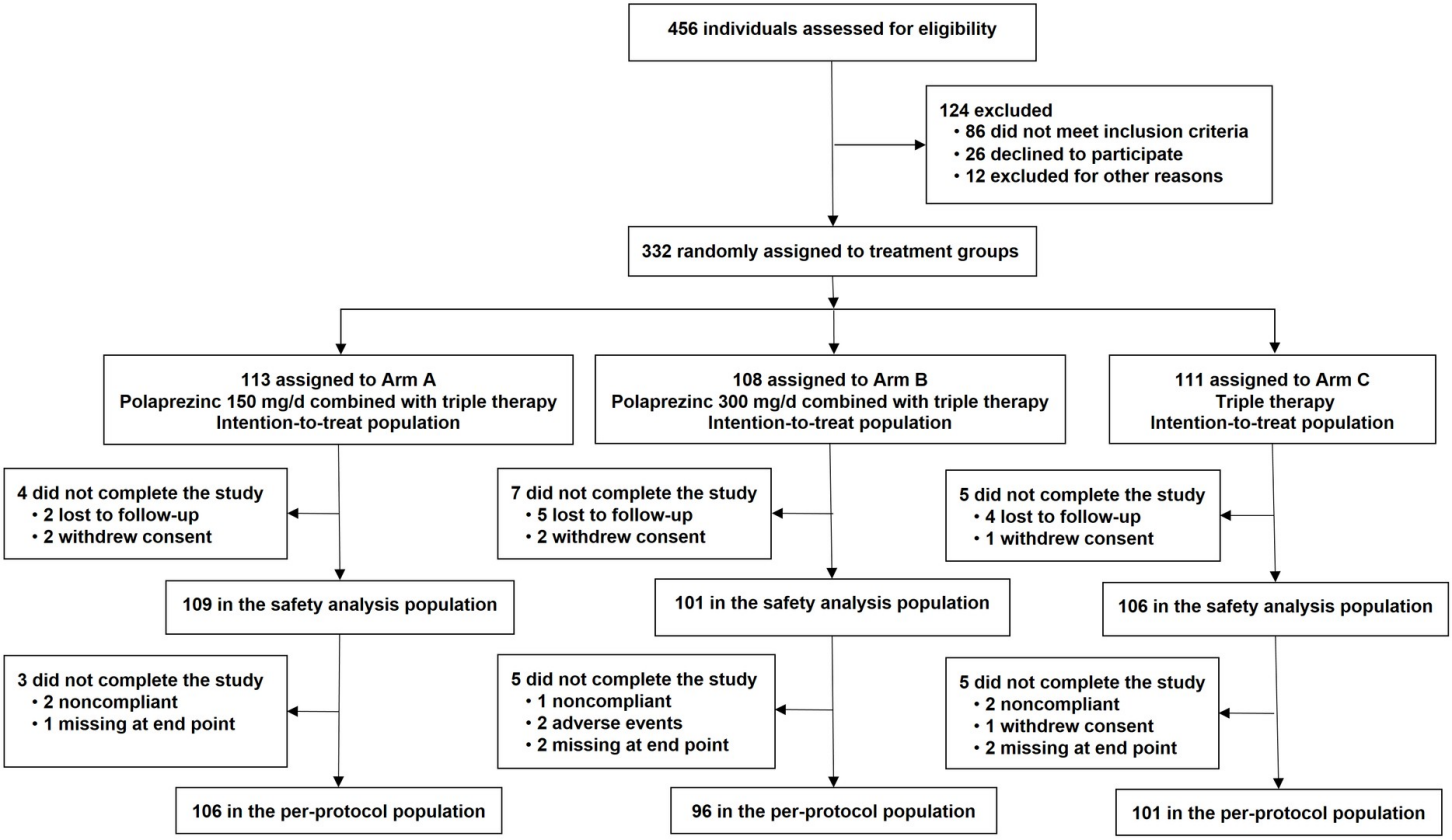
Participant flow.

Participant flow for this study is shown in Fig 1. From January 2014 to June 2015, 456 patients from 11 hospitals were screened for eligibility. Of these, 124 were excluded: 86 did not meet inclusion criteria, 26 declined to participate, and 12 were excluded for other reasons. 332 patients were enrolled and randomized into three groups for ITT analysis: 113 in Arm A (polaprezinc 150 mg/d combined with triple therapy), 108 in Arm B (polaprezinc 300 mg/d combined with triple therapy) and 111 in Arm C (triple therapy). 316 patients accepted the intervention and first-time safety assessment for the safety analysis set. 303 patients completed the study for PP analysis: 106 in Arm A, 96 in Arm B, and 101 in Arm C. The total dropout rate was 8.8%. No statistically significant differences were found between the three groups regarding baseline demographic data including age, gender, body mass index (BMI), and gastrointestinal symptoms in the ITT and PP populations (*P* > 0.05) (Table 1).

**Table 1:** Demographic data and baseline characteristics of participants.

	**ITT population**			***P* value**
	**Arm A**	**Arm B**	**Arm C**	
Number	113	108	111	
Age, mean ± SD (years)	41.0 ± 12.2	40.5 ± 13.6	41.0 ± 11.8	0.95
Gender (female/male)	65/48	50/58	53/58	0.19
BMI, mean ± SD (kg/m^2^)	22.56 ± 3.06	21.92 ± 4.16	23.02 ± 3.49	0.11
**Gastrointestinal symptoms at baseline**				
Upper abdominal pain[median (min, max)]	1.0 (0.0–3.0)	1.0 (0.0–2.0)	1.0 (0.0–3.0)	0.61
Acid reflux [median (min, max)]	0.0 (0.0–2.0)	0.0 (0.0–2.0)	0.0 (0.0–3.0)	0.87
Belching[median (min, max)]	0.0 (0.0–3.0)	0.0 (0.0–2.0)	0.0 (0.0–3.0)	0.72
Heartburn[median (min, max)]	0.0 (0.0–2.0)	0.0 (0.0–3.0)	0.0 (0.0–3.0)	0.34
Bloating[median (min, max)]	1.0 (0.0–2.0)	1.0 (0.0–2.0)	0.0 (0.0–3.0)	0.98
Nausea[median (min, max)]	0.0 (0.0–2.0)	0.0 (0.0–2.0)	0.0 (0.0–2.0)	0.23
Vomiting[median (min, max)]	0.0 (0.0–1.0)	0.0 (0.0–2.0)	0.0 (0.0–2.0)	0.25
	**PP population**			***P* value**
	**Arm A**	**Arm B**	**Arm C**	
Number	106	96	101	
Age, mean ± SD (years)	40.5 ± 12.2	40.1 ± 13.6	40.5 ± 11.9	0.97
Gender (female/male)	61/45	44/52	49/52	0.22
BMI, mean ± SD (kg/m^2^)	22.56 ± 3.11	22.00 ± 4.36	22.93 ± 3.48	0.23
**Gastrointestinal symptoms at baseline**				
Upper abdominal pain[median (min, max)]	1.0 (0.0–3.0)	1.0 (0.0–2.0)	1.0 (0.0–3.0)	0.72
Acid reflux[median (min, max)]	0.0 (0.0–2.0)	0.0 (0.0–2.0)	0.0 (0.0–3.0)	0.61
Belching[median (min, max)]	0.0 (0.0–3.0)	0.0 (0.0–2.0)	0.0 (0.0–3.0)	0.73
Heartburn[median (min, max)]	0.0 (0.0–2.0)	0.0 (0.0–2.0)	0.0 (0.0–3.0)	0.58
Bloating[median (min, max)]	1.0 (0.0–2.0)	1.0 (0.0–2.0)	0.0 (0.0–3.0)	0.94
Nausea[median (min, max)]	0.0 (0.0–2.0)	0.0 (0.0–2.0)	0.0 (0.0–2.0)	0.26
Vomiting[median (min, max)]	0.0 (0.0–1.0)	0.0 (0.0–1.0)	0.0 (0.0–2.0)	0.07

ITT, intention-to-treat; PP, per-protocol; BMI, body mass index; min, minimums; max, maximums.

Arm A, polaprezinc 150 mg/d combined with omeprazole, amoxicillin, clarithromycin for 14 days.

Arm B, polaprezinc 300 mg/d combined with omeprazole, amoxicillin, clarithromycin for 14 days.

Arm C, omeprazole, amoxicillin, and clarithromycin for 14 days.

### *H*. *pylori* eradication rates

**Fig 2:**
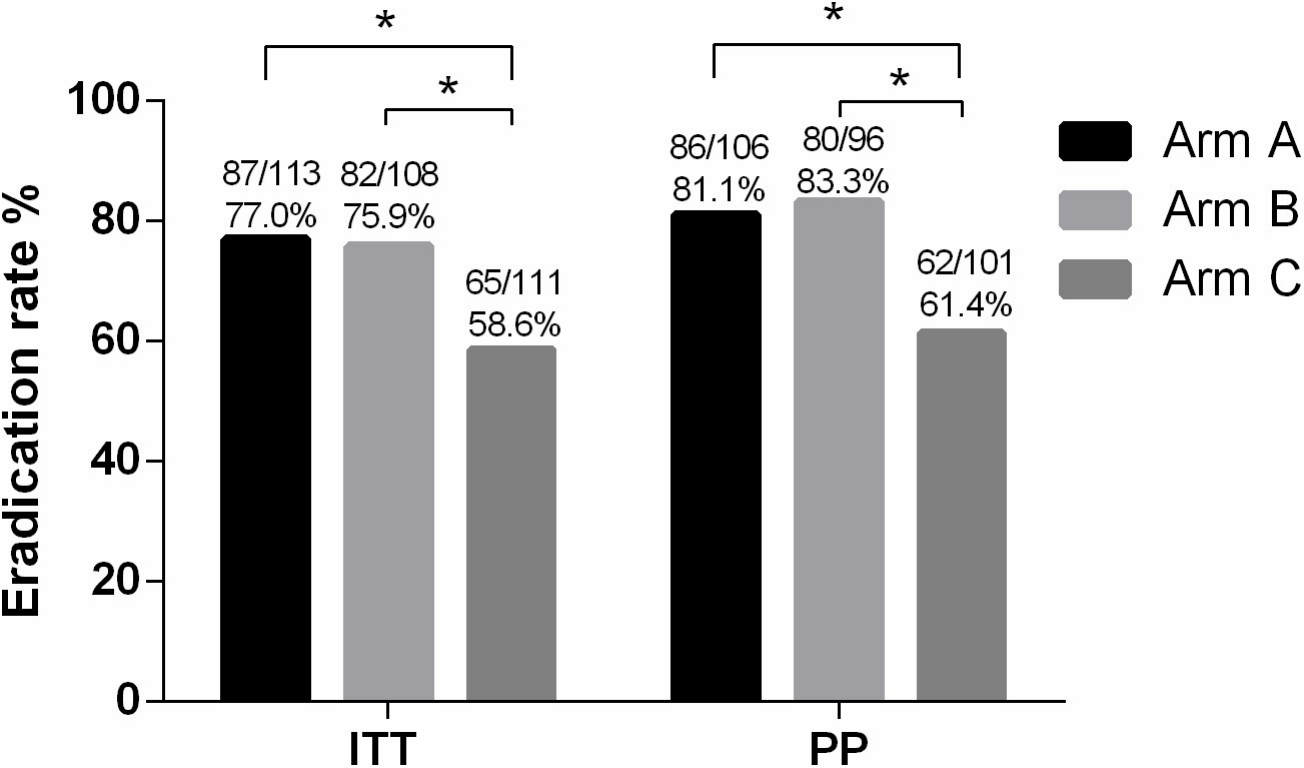
*H*. *pylori* eradication rates in the ITT and PP populations. ITT, intention-to-treat; PP, per-protocol Arm A, polaprezinc 150 mg/d combined omeprazole, amoxicillin, clarithromycin for 14 days Arm B, polaprezinc 300 mg/d combined omeprazole, amoxicillin, clarithromycin for 14 days Arm C, omeprazole, amoxicillin, and clarithromycin for 14 days *P < 0.01

The ITT analysis revealed *H*. *pylori* eradication rates of 77.0%, 75.9%, and 58.6% in Arms A, B, and C, respectively. The *H*. *pylori* eradication rate of polaprezinc combined with triple therapy was much higher than triple therapy alone, with 18.4% (*P* < 0.01, 95% CI [6.4–30.4]) improvement in Arm A and 17.4% (P < 0.01, 95% CI [5.2–29.6]) improvement in Arm B. There was no significant difference in *H*. *pylori* eradication rates between the two doses of polaprezinc– 150 mg/d (Arm A) and 300 mg/d (Arm B)–combined with triple therapy (*P =* 0.90) (Table 2, Fig 2).

**Table 2:** *H*. *pylori* eradication rates in the ITT and PP populations.

	ITT population		PP population	
*H*. *pylori* eradication rate: n/N (%)				
Arm A	87/113 (77.0%)		86/106 (81.1%)	
Arm B	82/108 (75.9%)		80/96 (83.3%)	
Arm C	65/111 (58.6%)		62/101 (61.4%)	
*H*. *pylori* eradication rate difference (%)				
Arm A vs Arm C	18.4%	*P* < 0.01,95% CI(6.4, 30.4)	19.7%	*P* < 0.01, 95% CI (7.7, 31.8)
Arm B vs Arm C	17.4%	*P* < 0.01,95% CI (5.2, 29.6)	21.9%	*P* < 0.01, 95% CI (9.9, 34.0)
Arm A vs Arm B	1.1%	*P* = 0.90,95% CI (-10.1, 12.3)	2.2%	*P* = 0.62, 95% CI (-12.7, 8.3)

ITT, intention-to-treat; PP, per-protocol.

Arm A, polaprezinc 150 mg/d combined with omeprazole, amoxicillin, clarithromycin for 14 days; Arm B, polaprezinc 300 mg/d combined with omeprazole, amoxicillin, clarithromycin for 14 days.

Arm C, omeprazole, amoxicillin, and clarithromycin for 14 days.

The PP analysis revealed *H*. *pylori* eradication rates of 81.1%, 83.3%, and 61.4% in Arms A, B, and C, respectively. The difference of *H*. *pylori* eradication rates between polaprezinc combined with triple therapy and triple therapy alone can further increase to reach 19.7% (Arm A vs. Arm C, *P* < 0.01, 95% CI [7.7–31.8]) and 21.9% (Arm B vs Arm C, *P* < 0.01, 95%CI [9.9–34.0]), respectively. There was no significant difference in the *H*. *pylori* eradication rates between the two polaprezinc doses combined with triple therapy (Arm A vs. Arm B) (*P* = 0.62) (Table 2, Fig 2).

### Symptom improvement

ITT and PP analyses evaluated the severity of gastrointestinal symptoms at day 7, 14, and 28 after treatment. Symptoms included abdominal pain, acid reflux, belching, heartburn, bloating, nausea, and vomiting. All three groups exhibited significant symptom improvement at day 7, 14, and 28 after treatment compared to baseline (*P* < 0.0001). However, there was no significant difference in symptom improvement between the three groups at day 7, 14, and 28 (*P* > 0.05) (Table 3).

**Table 3:** Symptom improvement in the ITT and PP populations.

Decrease in severity rate	ITT population				PP population			
	Arm A	Arm B	Arm C	*P* value	Arm A	Arm B	Arm C	*P* value
**N**	113	108	111		106	96	101	
**Abdominal pain**								
Day 7	52 (46.0%)	44 (40.7%)	40 (36.0%)	0.37	52 (49.1%)	43 (44.8%)	39 (38.6%)	0.35
Day 14	63 (55.8%)	54 (50.0%)	47 (42.3%)	0.29	61 (57.5%)	52 (54.2%)	47 (46.5%)	0.37
Day 28	63 (55.8%)	54 (50.0%)	54 (48.6%)	0.49	61 (57.5%)	52 (54.2%)	54(53.5%)	0.39
**Acid reflux**								
Day 7	34 (30.1%)	31 (28.7%)	28 (25.2%)	0.84	34 (32.1%)	27 (28.1%)	27 (26.7%)	0.74
Day 14	46 (40.7%)	36 (33.3%)	36 (32.4%)	0.83	46 (43.4%)	33 (34.4%)	35 (34.7%)	0.58
Day 28	47 (41.6%)	36 (33.3%)	33 (29.7%)	0.58	47 (44.3%)	34 (35.4%)	34 (33.7%)	0.51
**Belching**								
Day 7	25 (22.1%)	29 (26.9%)	28 (25.2%)	0.58	25 (23.6%)	27 (28.1%)	28 (27.7%)	0.77
Day 14	31 (27.4%)	35 (32.4%)	32 (28.8%)	0.60	31 (29.2%)	34 (35.4%)	32 (31.7%)	0.67
Day 28	32 (28.3%)	35 (32.4%)	36 (32.4%)	0.59	32 (30.2%)	34 (35.4%)	35 (34.7%)	0.64
**Heartburn**								
Day 7	28 (24.8%)	26 (24.1%)	24 (21.6%)	0.41	28 (26.4%)	23 (24.0%)	23 (22.8%)	0.96
Day 14	31 (27.4%)	31 (28.7%)	28 (25.2%)	0.50	31 (29.2%)	28 (29.2%)	26 (25.7%)	0.76
Day 28	31 (27.4%)	29 (26.9%)	29 (26.1%)	0.70	31 (29.2%)	27 (28.1%)	27 (26.7%)	0.82
**Bloating**								
Day 7	46 (40.7%)	38 (35.2%)	34 (30.6%)	0.82	46 (43.4%)	34 (35.4%)	33 (32.7%)	0.63
Day 14	54 (47.8%)	45 (41.7%)	41 (36.9%)	0.84	53 (50.0%)	42 (43.8%)	40 (39.6%)	0.71
Day 28	57 (50.4%)	47 (43.5%)	44 (39.6%)	0.88	56 (52.8%)	45 (46.9%)	42 (41.6%)	0.84
**Nausea**								
Day 7	22 (19.5%)	13 (12.0%)	17 (15.3%)	0.42	21 (19.8%)	12 (12.5%)	17 (16.8%)	0.40
Day 14	23 (20.4%)	16 (14.8%)	22 (19.8%)	0.55	22 (20.8%)	15 (15.6%)	21 (20.8%)	0.61
Day 28	24 (21.2%)	16 (14.8%)	23 (20.7%)	0.44	23 (21.7%)	15 (15.6%)	23 (22.8%)	0.44
**Vomiting**								
Day 7	10 (8.8%)	7 (6.5%)	14 (12.6%)	0.70	10 (9.4%)	5 (5.2%)	14 (13.9%)	0.16
Day 14	10 (8.8%)	7 (6.5%)	14 (12.6%)	0.70	10 (9.4%)	5 (5.2%)	14 (13.9%)	0.16
Day 28	10 (8.8%)	7 (6.5%)	14 (12.6%)	0.70	10 (9.4%)	5 (5.2%)	14 (13.9%)	0.16

ITT, intention-to-treat; PP, per-protocol.

Arm A, polaprezinc 150 mg/d combined with omeprazole, amoxicillin, clarithromycin for 14 days.

Arm B, polaprezinc 300 mg/d combined with omeprazole, amoxicillin, clarithromycin for 14 days.

Arm C, omeprazole, amoxicillin, and clarithromycin for 14 days.

### Adverse events

The incidence of adverse events was 3/107 (2.8%) in Arm A, 5/99 (5.1%) in Arm B, and 2/103 (1.9%) in Arm C. The incidence of adverse events in Arm B was much higher than in Arms A (*P* = 0.04) and C (*P* = 0.02). No participant experienced serious adverse events. One female participant in Arm B experienced increased salivation and involuntary shaking of the lower limbs on the second day of treatment. She discontinued treatment. Four weeks later, her alanine aminotransferase (ALT) level rose from 27 to 250 U/L and her aspartate aminotransferase (AST) level rose from 22 to 146 U/L. She was given oral polyene phosphatidylcholine and compound glycyrrhizin for 3 weeks, at which time her ALT and AST levels returned to normal. Another female patient complained of nonspecific symptoms including dizziness, palpitations, flushing, thirst, abdominal pain, numbness, and a gurgling sound after the first dose. This patient withdrew spontaneously and the symptoms promptly disappeared. Other adverse events included mild leukopenia, elevated liver enzymes or bilirubin, mildly elevated serum uric acid, and high blood pressure (Table 4).

**Table 4:** Adverse events in SAS population.

	SAS population		
	Arm A	Arm B	Arm C
SAS patients (n)	107	99	103
Adverse events	3 (2.8%)*	5 (5.1%)	2 (1.9%)*
Mild leukopenia	1 (0.9%)	0 (0.0%)	0 (0.0%)
Liver enzymes slightly elevated	2 (1.9%)	1 (1.0%)	1 (1.0%)
Bilirubin slightly elevated	0 (0.0%)	1 (1.0%)	0 (0.0%)
Serum uric acid mildly elevated	0 (0.0%)	1 (1.0%)	0 (0.0%)
Blood pressure slightly increased	0 (0.0%)	0 (0.0%)	1 (1.0%)
Nervous system symptoms	0 (0.0%)	1 (1.0%)^#^	0 (0.0%)
Nonspecific symptoms	0 (0.0%)	1 (1.0%)^†^	0 (0.0%)

SAS, safety analysis set.

Arm A, polaprezinc 150 mg/d combined with omeprazole, amoxicillin, clarithromycin for 14 days.

Arm B, polaprezinc 300 mg/d combined with omeprazole, amoxicillin, clarithromycin for 14 days.

Arm C, omeprazole, amoxicillin, and clarithromycin for 14 days.

*P<0.05 compared to Arm B.

^#^Participant in Arm B experienced increased salivation and involuntary shaking of the lower limbs on the second day of therapy. The participant discontinued treatment because of these adverse events.

†Participant in Arm B complained of nonspecific symptoms, including dizziness, palpitations, flushing, thirst, abdominal pain, numbness, and gurgling sound after the first dose. The participant withdrew spontaneously.

## Discussion

Our study shows that the mucosal protective agent polaprezinc when combined with clarithromycin-based triple therapy can significantly improve the rate of *H*. *pylori* eradication compared to standard clarithromycin-based triple therapy, without significant side effects. We showed that a higher dose of polaprezinc when combined with clarithromycin-based therapy did not further significantly increase efficacy. These results support the use of an adjuvant to improve the therapeutic efficacy of current anti–*H*. *pylori* treatment regimens, even in areas of high antibiotic resistance.

Polaprezinc is a chelate compound consisting of 23% zinc and 77% l-carnosine. The typical clinical oral dose is 150 mg/d, which contains 34 mg zinc and 116 mg l-carnosine [8]. Its pharmacological activity is attributable mainly to the zinc ion [8]. The exact mechanism by which polaprezinc enhances anti–*H*. *pylori* therapy is unclear. Previous studies have shown that zinc chloride inhibits *H*. *pylori* growth and reduces the expression of interleukin 1 beta (IL-1β) by gastric epithelial cells [9]. Spectrophotometric examination has revealed that polaprezinc scavenges monochloramine in *H*. *pylori*–infected Mongolian gerbils [10]. Some studies have shown that the zinc complex with famotidine can inhibit both the activity of the human urease enzyme and the growth of *H*. *pylori*; the anti-*H*. *pylori* activity observed was comparable in antibiotic-resistant and antibiotic-sensitive strains [11]. Another potential mechanism is that zinc replaces the nickel ions at the urease active site, which interferes with the complex-forming ability of the two metal ions and results in inhibition of urease activity and growth retardation of *H*. *pylori* [12].

Polaprezinc has been used as an effective anti-ulcer drug in Japan for more than 20 years. It is also used to reduce small intestine mucosal injury from low-dose aspirin [13], accelerate healing of pressure ulcers [14], and prevent oral mucositis associated with radiotherapy [15]. Polaprezinc had previously shown promise as a gastric mucosal protective agent in combination with triple therapy to improve the rate of *H*. *pylori* eradication. Yakoob and colleagues first demonstrated the susceptibility of *H*. *pylori* to zinc chloride in 116 clinical isolates of *H*. *pylori*; and further showed that the susceptibility of these strains to zinc chloride 40 μg/mL or bismuth subsalicylate 20 μg/mL was similar– 95% vs 98% in vitro [16]. Subsequently, Kuwayama and colleagues reported that polaprezinc in combination with low-dose metronidazole (750 mg/d) and amoxicillin (750 mg/d) completely eradicated *H pylori* [17]. Moreover, Kashimura and colleagues found that polaprezinc combined with triple therapy can significantly improve *H*. *pylori* eradication rates from 77.4% to 94.3% [7].

Our study confirmed that 75 mg twice daily polaprezinc combined with triple therapy significantly increases the *H*. *pylori* eradication rate to 77% (ITT)– 81.1% (PP), much higher than traditional triple therapy at 58.6% (ITT)– 61.4% (PP). Increasing the twice daily dose of polaprezinc from 75 mg to 150 mg does not further improve the clinical outcome. The present high resistance rates to clarithromycin (37.5%), metronidazole (67.2%), and levofloxacin (33.5%) have made empiric treatment difficult in Chinese patient populations [18]. Although the resistance rates of amoxicillin (6.8%), tetracycline (3.5%), rifampicin (14.2%), and furazolidone are low, their side effects and limited availability have restricted their use [18]. Tailored therapy is generally unavailable in China, and *H*. *pylori* culture and antibiotic susceptibility tests are invasive and costly, as a biopsy specimen must be obtained during gastroscopy. Our study showed that in regions with high rates of antibiotic resistance, *H*. *pylori* eradication rates can be greatly improved by combining a safe mucosal protective agent with standard triple therapy.

This study also showed significant improvement in the severity of gastrointestinal symptoms at 7, 14, and 28 days after eradication therapy in all groups, compared to baseline symptoms. However, polaprezinc combined with triple therapy did not show an advantage compared to triple therapy alone. This is likely due to the short duration of follow-up. We would propose extending the follow-up period to 6 to 12 months to compare differences in symptoms.

Lastly, we observed a higher incidence of adverse events in the high-dose polaprezinc group compared to the standard dose polaprezinc or triple therapy alone groups. Among the high-dose polaprezinc combined with triple therapy group, one participant experienced excessive salivary secretion and involuntary shaking of the lower limbs on the second day of therapy, along with a transient elevation of liver enzymes. These adverse events were likely due to the toxic effect of the high zinc concentration when polaprezinc is used at a high dose, but may also be related to individual drug sensitivity. Other rare side effects, including mild leukopenia, elevated liver enzymes, elevated serum uric acid, and high blood pressure were inconsequential and normalized after completion of the study. Overall, the standard dose of polaprezinc when combined with clarithromycin-based triple therapy was shown to be safe and well tolerated.

Our study has several limitations. First, patients with a positive ^13^C or ^14^C urea breath test and without prior eradication therapy were enrolled in this study. We did not use a second non-invasive diagnostic method to confirm the *H*.*pylori* infection, because, at the time of our study, *H*.*pylori* serology kits with high local validation and *H*.*pylori* stool antigen tests were not widely available in China. Second, bismuth-containing quadruple therapy for 14 days is currently the first-line treatment in China due to the high dual resistance of clarithromycin and metronidazole [4]. Noninferiority comparison trials to assess the relative merits of polaprezinc-containing versus bismuth-containing quadruple therapy are needed. A previous randomized, open-label, multicenter prospective study reported a bismuth-containing quadruple therapy *H*. *pylori* eradication rate of 68% (ITT)– 77% (PP) in China [6], and sequential and concomitant therapy eradication rates of 72.1% (ITT)– 76.5% (PP) [19] and 78.3% (ITT)– 87.4% (PP) [20], respectively. Also, in vitro susceptibility of *H*. *pylori* clinical isolates to zinc chloride 40 μg/mL was similar to bismuth subsalicylate 20 μg/mL (95% vs 98%) [16]. Thus, we believe that polaprezinc-containing quadruple therapy with a 77.0% (ITT)– 81.1% (PP) eradication rate would obtain similar or better results in future comparisons to bismuth-containing quadruple therapy, sequential therapy, or concomitant therapy. Further, polaprezinc-containing quadruple therapy may achieve higher compliance than sequential therapy, and it requires fewer antibiotics than concomitant therapy. Third, we compared the triple therapy of omeprazole, amoxicillin and clarithromycin, with and without the addition of polaprezinc, for the treatment of *H*. *pylori* infections. Resistance to amoxicillin is rare; however, resistance to clarithromycin is a critical factor. We regret that we did not determine the clarithromycin resistance in order to predict the eradication rate in other populations with known clarithromycin resistance, using the Hp-normogram [21], which is based on the eradication rates in both clarithromycin-sensitive and clarithromycin-resistant patients.

Overall, we confirmed the effectiveness of the zinc compound, polaprezinc, in improving the *H*. *pylori* eradication rate, and we verified the results of the prior small study in Japan [7]. Acknowledging that the improvement in the eradication rate was less than ideal in the current study, we intend to conduct the head-to-head comparison of polaprezinc-containing quadruple therapy and bismuth-containing quadruple therapy, with the testing of clarithromycin resistance. We will replace omeprazole with rabeprazole to increase acid inhibition, which may further improve the eradication rate. We will also investigate the mechanisms by which polaprezinc enhances the rate of *H*. *pylori* eradication when combined with triple therapy.

## Supporting information

S1 File The CONSORT Checklist. (DOC)

S2 File The protocol in Chinese. (DOC)

S3 File The protocol in English. (DOCX)

S4 File The primary ethical approval in Chinese. (PDF)

S5 File The translation of primary ethical approval in English. (PDF)

S1 Table The data set of clinical trial result. (XLSX)
