# Supplementary material for: Polaprezinc combined with clarithromycin-based triple therapy for Helicobacter pylori-associated gastritis: A prospective, multicenter, randomized clinical trial
Source: PLoS One. 2017 Apr 13;12(4):e0175625. doi: 10.1371/journal.pone.0175625 (PMC5391070; doi:10.1371/journal.pone.0175625)
Supplement: S2 File — (DOC) [file pone.0175625.s002.doc]

**三联疗法联合聚普瑞锌（3+P疗法）治疗幽门螺杆菌相关性胃炎多中心、随机、平行对照临床研究**

**参加单位 负责人**

**负责单位： 北京协和医院 钱家鸣**

协作单位：

| **上海交通大学医学院附属仁济医院** | **房静远** |  |
| --- | --- | --- |
| **山东大学齐鲁医院** | **李延青** |  |
| **郑州医科大学第一附属医院** | **李建生** |  |
| **南昌大学附属第一医院** | **吕农华** |  |
| **武汉大学人民医院** | **罗和生** |  |
| **中南大学湘雅医院** | **邹益友** |  |
| **浙江大学附属第一医院** | **季 峰** |  |
| **吉林大学第一临床医院** | **徐 红** |  |
| **厦门大学附属中山医院** | **任建林** |  |
| **南京医科大学第一附属医院** | **施瑞华** |  |
|  |  |  |
|  |  |  |
|  |  |  |

方案摘要

| 试验药物名称 | 聚普瑞锌颗粒 |
| --- | --- |
| 研究题目 | 三联疗法联合聚普瑞锌（3+P疗法）治疗幽门螺杆菌相关性胃炎多中心、随机、平行对照临床研究 |
| 试验目的 | 评价聚普瑞锌颗粒联合三联方案用于提高Hp根除率临床有效性 |
| 试验设计 | 随机、平行对照、多中心试验 |
| 纳入标准 | 年龄在18～70岁；C13/C14呼气试验Hp阳性；Hp阳性初治患者；半年内胃镜检查诊断为胃炎，并排除有胃溃疡、胃癌等胃部重大疾病患者；青霉素皮试阴性；签署知情同意书。 |
| 有效性评价指标 | Hp根除率 |
| 安全性评价指标 | 1.聚普瑞锌可能出现的不良反应症状。肝功能损伤、皮疹、瘙痒、荨麻疹、嗜睡、乏力，便秘、嗳气、腹胀、呕吐、腹泻等。  2.奥美拉唑、克拉霉素、阿莫西林三联疗法常见不良反应为头痛、腹泻、恶心、呕吐、皮疹、哮喘、假膜性肠炎、贫血、血小板减少、嗜酸性粒细胞增多、味觉异常和肝转氨酶短暂升高、血清氨基转移酶可轻度增高、便秘、听觉障碍等。  3.生命体征：包括体温、脉搏、呼吸、血压等。  4.常规检查：血常规、尿常规、心电图、肾功能、肝功能。 |
| 受试者数量 | 治疗组a：110例；治疗组b：110例；对照组：110例；  包括20%可能脱落的病例，合计330例。 |
| 给药方案 | 对照组：三联疗法14天，奥美拉唑（洛赛克）+阿莫西林+克拉霉素  治疗组a：三联疗法联合聚普瑞锌（3+P疗法）14天，奥美拉唑（洛赛克）+阿莫西林+克拉霉素+聚普瑞锌（75mg）  治疗组b：三联疗法联合聚普瑞锌（3+P疗法）14天，奥美拉唑（洛赛克）+阿莫西林+克拉霉素+聚普瑞锌（150mg） |
| 疗程 | 2周 |
| 试验进度安排 | 预计6个月内完成试验 |

**一、诚信申明**

本研究保证严格按照试验规程进行并确保数据记录的真实性，无利益冲突。

**二、研究题目：**三联疗法联合聚普瑞锌（3+P疗法）治疗幽门螺杆菌相关性胃炎多中心、随机、平行对照临床研究

**三、研究计划书版本号：**1.0

**四、研究经费来源：**吉林省博大伟业制药有限公司

**五、研究事项执行流程图**

**试验流程表**

| 项 目 | 治疗前 | 治疗过程 | | 疗程结束后 |
| --- | --- | --- | --- | --- |
|  | 0周（-5～-1天） | 1周末 | 2周末 | 6-8周内 |
| 筛 选 | × |  |  |  |
| 签署知情同意书 | × |  |  |  |
| 确定纳入排除标准 | × |  |  |  |
| 病史采集 | × |  |  |  |
| 生命体征 | × | × | × | × |
| 随机分组 | × |  |  |  |
| 血常规检查 | × |  |  | × |
| 尿常规检查 | × |  |  | × |
| 肾功能检查 | × |  |  | × |
| 肝功能检查 | × |  |  | × |
| 妊娠检查（生育期妇女） | × |  |  |  |
| 心电图 | × |  |  | × |
| C13/C14尿素呼气试验 | × |  |  | × |
| 治疗与随访 |  | × | × | × |
| 合并用药 | × | × | × | × |
| 记录不良反应 |  | × | × | × |
| 回收剩余药品 |  |  | × |  |

注: “×”为必须按随访时间检查的项目或操作内容。

**六、研究背景**

幽门螺杆菌（[Helicobacter pylori](http://www.baidu.com/link?url=bsYg435LHs_dskY3oXKzg0VdEOj4I5f452TBEdXFcZsHcZcpSPcEvJECvLqx_9r9gSNu7Fn-NAu0rK7kCHFON4y1wMlAngAfOTGXu4qfDXx32wX36ibAeU4b-ifyd3HR), H.pylori）早自1994年起即被世界卫生组织列为胃癌一级致癌因素^[1]^，根除H.pylori可以减少未来胃癌发生率^[2]^。H.pylori在我国成人中感染率高达40%-60%，国内外指南中^[3-4]^均推荐消化性溃疡、胃黏膜相关淋巴组织淋巴瘤、服用非甾体消炎药、功能性消化不良、慢性胃炎伴糜烂萎缩、胃癌患者一级亲属、胃肿瘤已行内镜下或手术治疗、长期服用PPI、不明原因缺铁性贫血、特发性血小板减少性紫癜、个人积极要求治疗者均需考虑根除H.pylori治疗。尤其是我国是胃癌高发国家，根除H.pylori可以降低胃癌发生率，Take等^[2]^通过对1222例消化性溃疡患者进行根除H.pylori治疗后，H.pylori根除失败组患者随访中胃癌发生率高于H.pylori根除成功组(0.45%/年VS0.21%/年，*P*=0.049)。根除成功组中胃癌发生最长间隔是14.5年，而根除失败组为13.7年。

推荐用于根除H.pylori治疗的抗菌药物中，甲硝唑耐药率达到60%-70%，克拉霉素达到20%-38%，左氧氟沙星达到30-38%,阿莫西林、呋喃唑酮和四环素耐药率尚仍较低1%-5%。伴随H.pylori耐药率升高，标准三联疗法PPI+克拉霉素+阿莫西林治疗H.pylori根除率已低于或远低于80%，疗程从7天延长至10天，根除率仅能提高5%。国际上推荐的序贯疗法、伴同疗法、和左氧氟沙星三联疗法在我国均未表现出明显优势^[3]^。目前在我国首先推荐含铋剂的四联疗法，但长期服用铋剂存在一定不良反应，包括神经毒性、肾功能损伤、牙齿着色以及消化道症状如恶心呕吐、粪色变黑等。我国指南中亦提到部分胃黏膜保护剂可替代铋剂用于四联疗法获得相同疗效^[5]^。

聚普瑞锌是微量元素锌和L-肌肽的络合物，具有抗氧化作用的粘膜保护剂，促进消化性溃疡的愈合^[6]^，推荐口服剂量75mg 2次/日。动物实验证实聚普瑞锌能够诱导损伤后大鼠胃黏膜产生大量热休克蛋白70发挥保护胃黏膜作用^[7]^。同时，聚普瑞锌通过阻断H.pylori定植；抑制尿素酶活性；清除尿素酶及一氯胺^[8]^；抑制相关炎症因子以提高幽门螺杆菌清除率。已有动物实验表明，聚普瑞锌可以通过抑制多形核白细胞表达的CD11b/CD18以及胃上皮细胞产生的IL-8等明显减轻H.pylori相关性胃炎炎症反应^[9-10]^。Kashimura等研究表明，聚普瑞锌联合三联疗法兰索拉唑、阿莫西林、克拉霉素，可将H.pylori根除率由24/31（77.4%）提升至33/35（94.3%），且两组患者不良反应发生率无明显差异^[11]^。

中国既是H.pylori的高感染国家，又是抗生素耐药率高的国家，抗生素耐药率高也是伴同疗法在中国失败的主要原因，寻找安全有效提高根除H.pylori的疗法迫在眉睫。本研究将基于前期动物实验和日本小规模单中心研究基础上，在我国从南至北联合11家三级甲等重点医院，进行本项大规模随机、平行对照、多中心前瞻性临床研究，进一步评估不同剂量聚普瑞锌联合三联疗法治疗幽门螺杆菌的有效性和安全性的评估。

**七、研究目的**

评估不同剂量聚普瑞锌联合三联疗法治疗幽门螺杆菌的有效性和安全性的评估

**八、研究设计：**本研究为全国多中心、随机、平行对照性的临床研究

**1、纳入和排除标准**

A. 入选标准：

1. 半年内有过胃镜检查诊断为胃炎，并排除有胃溃疡、胃癌等胃部重大疾病患者。
2. C13/C14尿素呼气试验Hp阳性者。
3. 年龄18～70岁，男女不限。
4. Hp阳性初治患者。
5. 青霉素皮试无过敏者。
6. 签署知情同意书。

B. 排除标准：

1. 治疗前4周用过抗生素、铋剂或者治疗前2周用过PPI者。
2. 妊娠或哺乳期妇女。
3. 存在其它影响本研究评价的严重疾病如严重的肝病、心脏病、肾脏病、恶性肿瘤及酒精中毒疾患。
4. 入组前3个月内参加过其它临床试验者。
5. 对本研究所用药物过敏者，或既往有青霉素过敏史者。
6. 不能正确表达自己主诉，如精神病、严重神经官能症，不能合作本试验者。

**2. 设计方案、设计模式图**

随机、平行对照、多中心、前瞻性临床试验（优效）


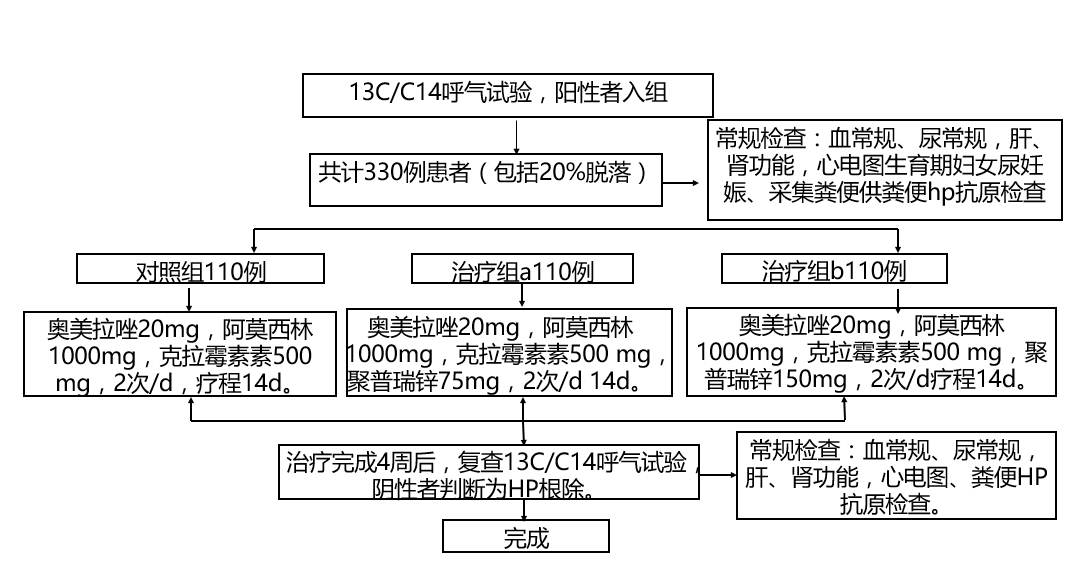


**3. 参试者的征募**

自2014年1月至2015年6月就诊于11家医院消化科门诊的患者，经C13或C14呼气试验检测和胃镜检查证实幽门螺杆菌相关性胃炎患者，获取知情同意后，根据入选和排除标准进行筛选，并完善体格检查和实验室检查。

**4. 参试者一般信息的收集**

收集受试者一般信息包括性别、年龄、身高、体重、既往史、药物过敏史等。

**5. 基线指标和观测项目**

基线指标包括体格检查（生命体征）、实验室检查（血常规、生化全项、尿常规、心电图）和消化道症状自我评测分数。

**6.试验分组**

入选总数：330例，随机分为以下3组：每组110例。

（1）对照组：三联疗法14天，奥美拉唑（洛赛克）+阿莫西林+克拉霉素

（2）治疗组a：三联疗法联合聚普瑞锌（3+P疗法）14天，奥美拉唑（洛赛克）+阿莫西林+克拉霉素+聚普瑞锌（75mg）

（3）治疗组b：三联疗法联合聚普瑞锌（3+P疗法）14天，奥美拉唑（洛赛克）+阿莫西林+克拉霉素+聚普瑞锌（150mg）

| 对照组 | 疗程 | 治疗组 | 疗程 |
| --- | --- | --- | --- |
| 洛赛克 20mg bid | 14天 | 洛赛克 20mg bid | 14天 |
| 阿莫西林 1000mg bid |  | 阿莫西林 1000mg bid |  |
| 克拉霉素 500mg bid |  | 克拉霉素 500mg bid |  |
|  |  | 聚普瑞锌75mg/150mg bid |  |

**7.药品构成**

洛赛克 阿斯利康制药有限公司

阿莫西林（阿莫仙） 联邦制药

克拉霉素 西安利君制药有限责任公司

聚普瑞锌 吉林省博大伟业制药有限公司

**8. 样本量估算**

根据主要研究终点幽门螺杆菌根除率估算样本量。先前小样本量研究表明聚普瑞锌联合三联疗法可将幽门螺杆菌根除率由77%提高到94%[11]，采用双侧α=0.05，β=0.20，检测15%差异性，估算样本量为每组112名患者。

**9. 随机和隐藏分组方法**

北京协和医学院流行病学和卫生统计学系设计并提供给各个研究中心随机表，研究者提供新入选受试者姓名和序号，由另一名独立研究者根据序号，按照随机表随机入组至试验组A、试验组B和对照组。此名独立研究者负责分发和回收药物。

**10. 盲法：**基于本试验主要终点为客观指标，为开放性研究

**11. 疗效评价**

（1）主要疗效指标：各组停药4周后Hp根除率。
（2）次要疗效指标：疗程结束后的症状缓解率（详见患者登记表）和安全性评估。
Hp感染及根除诊断方法

1、Hp感染诊断方法：凡C13尿素呼气试验（≥4.0±0.4）/C14尿素呼气试验（^14^C-UBT≥100dpm/mmolCO_2_）阳性者可判断为Hp感染。

2、Hp根除判断方法：患者停药4周后，进行C13/C14尿素呼气试验，阴性者判断为Hp根除。（检测试剂采用海得威公司的试剂）。

（3）安全性评价：试验期间所发生的不良事件（包括严重不良事件），应根据其严重程度及时准确记录，并判定与受试药物之间的关系，并通过统计学数据来评估安全性。

**12、不良事件和不良反应的定义、鉴定方法和管理制度**

（1）不良事件定义 ：自患者签署知情同意书并入选研究开始至最后一次随访之间，由病人报告或研究医师报告的任何不利的医疗事件均应记录在案，无论与研究药物是否有因果关系，均判定为不良事件。治疗后任何体格检查、实验室检查的异常改变均应作为不良事件记录。

（2）不良事件记录：研究期间应如实记录不良事件的发生时间、与研究药物关系、严重程度、持续时间、采取的措施和转归。不良事件应记录在指定的CRF不良事件报告表中。

（3）不良事件严重程度判定标准：在填写CRF的不良事件表时，研究者将使用轻度、中度、重度来描述不良事件的强度。为统一标准，事件强度的分级如下：

轻度：不影响受试者的正常功能；

中度：一定程度上影响到受试者的正常功能；

重度：明显影响受试者的正常功能。

注意区别不良事件的严重程度和强度。重度用来描述强度，不一定是SAE。例如头痛可能在强度上表现为重度，但不能列入SAE，除非它符合SAE标准。

（4）不良事件与研究用药关系的判断标准

研究者应对不良事件和研究药物以及合并用药之间可能存在的关联作出评估，请根据以下原则判断该不良事件与研究药物的因果关系：

4.1开始用药的时间和不良事件出现的时间有无合理的先后关系；

4.2所怀疑的不良事件是否符合该药物已知的不良反应类型；

4.3 所怀疑的不良事件是否可用合并用药的作用、病人的临床状态或其他疗法的影响来解释；

4.4 停药或减量后，不良事件是否消失或减轻。

4.5 再次服用药物是否出现同样的反应。

根据以上原则，将因果关系分为肯定有关，很可能有关，可能有关，可能无关和无关5个等级。

|  | 1 | 2 | 3 | 4 | 5 |
| --- | --- | --- | --- | --- | --- |
| 肯定有关 | ＋ | ＋ | － | ＋ | ＋ |
| 很可能有关 | ＋ | ＋ | － | ＋ | ？ |
| 可能有关 | ＋ | ＋ | ± | ± | ？ |
| 可能无关 | ＋ | － | ± | ± | ？ |
| 无关 | － | － | ＋ | － | － |

注：＋表示肯定，－表示否定，±表示难以肯定或否定，？表示情况不明

（5）严重不良事件

5.1 严重不良事件的判定

不良事件符合下面一条或一条以上标准时归为严重不良事件（SAE）：

- 死亡
- 有生命危险（如：有马上死亡的危险）
- 导致住院治疗或住院时间延长
- 永久或严重致残
- 先天畸形或缺陷
- 有些还没有导致死亡、生命危险或需住院的医疗事件，经研究医师判断，认为其可能对病人造成危害或需药物或外科手术治疗以避免上述情况的发生，也应视为SAE。

5.2 发生严重不良事件时对病例的处理

患者应退出试验并接受相应治疗。

5.3 严重不良事件的记录与报告

临床研究过程中的任何严重不良事件，必须立即报告监视员和北京协和医院药物临床研究机构，由机构上报给伦理委员会。同时，研究者必须填写严重不良事件报告表，记录严重不良事件的发生时间、严重程度、持续时间、采取的措施和转归。

如病人在试验期间内突然死亡，除以严重不良事件报告外，如有尸检结果还应附送尸检报告单。

**13、统计分析方法**

研究主要终点Hp根除率和次要终点消化道症状改善率采用ITT(intention-to-treat)数据集和PP(per-protocol)数据集进行分析，次要终点副作用采用SAS(Safety Analysis Set)分析。ITT数据集是指所有经随机化分组，并至少使用一次药品的全部病例构成，，其中缺失数据用本周期最后一次观察的数据填补（LOCF准则）为保守估计疗效。PP数据集是指所有依从性好并完成全部试验的患者。SAS数据集是指所有经随机化分组，有一次研究药物并进行了至少一次安全性评估的患者，构成的安全性人群。对于计量资料，符合参数检验条件的采用方差分析方法；对于不符合参数检验条件的计量资料和等级资料采用多组秩和检验完成分析。对于计数资料符合χ2检验条件的采用χ2检验，不符合χ2检验条件的行精确概率法检验。对于Hp根除率，采用按中心分层的CMH-χ2检验进行组间比较。并采用Mantel-Haensze法计算每组有效率的95%可信区间，及组间根除率差值的95%可信区间。统计分析将采用SAS 9.3统计分析软件进行计算。所有的统计检验均采用双侧检验，P值小于或等于0.05将被认为所检验的差别有统计意义。由北京协和医学院流行病学和卫生统计学系完成。

**14、参试者管理制度**

患者如出现下列情况退出研究：（1）病情恶化或出现严重并发症；（2）试验期间出现严重副作用，病人无法耐受者；（3）治疗期间病人出现其它疾病干扰本观察；（4）失访；（5）治疗期间妊娠。

**15、标本管理制度**

本研究中患者按照临床检验流程，在医院采血窗口进行采血后送检血常规、生化全项，留取尿液标本进行尿常规，由检验科按照正常标本流程处理标本。

**16、药品和器材管理制度**

由独立研究者（双人签字核对）统一保管储存、分发核对、回收药品。

**17、数据管理制度**

由独立研究者根据CRF表和患者日志统一录入数据统一保管，并送北京协和医学院流行病学和卫生统计学系进行统计分析。

**18、试验结束后受试者的治疗和管理：**对试验结束后未能根除幽门螺杆菌患者予以随访，根据患者意愿和治疗情况，必要时于休疗2-3月后再次进行根除幽门螺杆菌治疗

**19、研究团队**

| 北京协和医院 | 钱家鸣 | 主要研究负责者 |
| --- | --- | --- |
| 上海交通大学医学院附属仁济医院 | 房静远 | 分中心研究负责者 |
| 山东大学齐鲁医院 | 李延青 | 分中心研究负责者 |
| 郑州医科大学第一附属医院 | 李建生 | 分中心研究负责者 |
| 南昌大学附属第一医院 | 吕农华 | 分中心研究负责者 |
| 武汉大学人民医院 | 罗和生 | 分中心研究负责者 |
| 中南大学湘雅医院 | 邹益友 | 分中心研究负责者 |
| 浙江大学附属第一医院 | 季 峰 | 分中心研究负责者 |
| 吉林大学第一临床医院 | 徐 红 | 分中心研究负责者 |
| 厦门大学附属中山医院 | 任建林 | 分中心研究负责者 |
| 南京医科大学第一附属医院 | 施瑞华 | 分中心研究负责者 |

**参考文献**

[1] Schistosomes, liver flukes and Helicobacter pylori. IARC Working Group on the Evaluation of Carcinogenic Risks to Humans, Lyon, 7–14 June 1994. IARC Monogr Eval Carcinog Risks Hum. 1994;61:1–241.

[2] [Take S](http://www.ncbi.nlm.nih.gov/pubmed/?term=Take S%5BAuthor%5D&cauthor=true&cauthor_uid=25351555), [Mizuno M](http://www.ncbi.nlm.nih.gov/pubmed/?term=Mizuno M%5BAuthor%5D&cauthor=true&cauthor_uid=25351555), [Ishiki K](http://www.ncbi.nlm.nih.gov/pubmed/?term=Ishiki K%5BAuthor%5D&cauthor=true&cauthor_uid=25351555),et al. Seventeen-year effects of eradicating Helicobacter pylori on the prevention of gastric cancer in patients with peptic ulcer; a prospective cohort study. [J Gastroenterol.](http://www.ncbi.nlm.nih.gov/pubmed/25351555) 2015;50(6):638-644. PMID:25351555

[3]中华医学会消化病学分会幽门螺杆菌学组/全国幽门螺杆菌研究协作组.第四次全国幽门螺杆菌感染处理共识报告.中华内科杂志,2012;10(51):832-837.

[4] Malfertheiner P, Megraud F, O'Morain CA, et al.Management of Helicobacter pylori infection--the Maastricht IV/ Florence Consensus Report. Gut. 2012 ;61(5):646-664. PMID:22491499

[5] 梁洁,吴开春,杨云生等. 依卡倍特钠四联疗法根除幽门螺杆荫的临床试验：全国多中心临床研究．中华消化杂志，2012;10(32):662-664.

[6] [Matsukura T](http://www.ncbi.nlm.nih.gov/pubmed/?term=Matsukura T%5BAuthor%5D&cauthor=true&cauthor_uid=10951100), [Tanaka H](http://www.ncbi.nlm.nih.gov/pubmed/?term=Tanaka H%5BAuthor%5D&cauthor=true&cauthor_uid=10951100). Applicability of zinc complex of L-carnosine for medical use. [Biochemistry.](http://www.ncbi.nlm.nih.gov/pubmed/10951100) 2000; 65(7):817-823. PMID:10951100

[7] 杨晓鸥,钱家鸣,陈蔷.聚普瑞锌诱导HSP70保护大鼠胃黏膜损伤.胃肠病学和肝病学杂志.2014; 1(23):50-53.

[8] [Ishihara R](http://www.ncbi.nlm.nih.gov/pubmed/?term=Ishihara R%5BAuthor%5D&cauthor=true&cauthor_uid=12485126), [Iishi H](http://www.ncbi.nlm.nih.gov/pubmed/?term=Iishi H%5BAuthor%5D&cauthor=true&cauthor_uid=12485126), [Sakai N](http://www.ncbi.nlm.nih.gov/pubmed/?term=Sakai N%5BAuthor%5D&cauthor=true&cauthor_uid=12485126), et al. Polaprezinc attenuates Helicobacter pylori-associated gastritis in Mongolian gerbils. [Helicobacter.](http://www.ncbi.nlm.nih.gov/pubmed/12485126) 2002; 7(6):384-389. PMID:12485126

[9] [Handa O](http://www.ncbi.nlm.nih.gov/pubmed/?term=Handa O%5BAuthor%5D&cauthor=true&cauthor_uid=12464972), [Yoshida N](http://www.ncbi.nlm.nih.gov/pubmed/?term=Yoshida N%5BAuthor%5D&cauthor=true&cauthor_uid=12464972), [Tanaka Y](http://www.ncbi.nlm.nih.gov/pubmed/?term=Tanaka Y%5BAuthor%5D&cauthor=true&cauthor_uid=12464972),et al. Inhibitory effect of polaprezinc on the inflammatory response to Helicobacter pylori. [Can J Gastroenterol.](http://www.ncbi.nlm.nih.gov/pubmed/12464972) 2002; 16(11):785-789. PMID:12464972

[10] [Suzuki H](http://www.ncbi.nlm.nih.gov/pubmed/?term=Suzuki H%5BAuthor%5D&cauthor=true&cauthor_uid=11328267), [Mori M](http://www.ncbi.nlm.nih.gov/pubmed/?term=Mori M%5BAuthor%5D&cauthor=true&cauthor_uid=11328267), [Seto K](http://www.ncbi.nlm.nih.gov/pubmed/?term=Seto K%5BAuthor%5D&cauthor=true&cauthor_uid=11328267), et al. Polaprezinc attenuates the Helicobacter pylori-induced gastric mucosal leucocyte activation in Mongolian gerbils--a study using intravital videomicroscopy. [Aliment Pharmacol Ther.](http://www.ncbi.nlm.nih.gov/pubmed/11328267) 2001;15(5):715-725. PMID:11328267

[11] Kashimura H, Suzuki K, Hassan M,et al. Polaprezinc, a mucosal protective agent, in combination with lansoprazole, amoxicillin and clarithromycin increases the cure rate of Helicobacter pylori infection. Aliment Pharmacol Ther. 1999;13:483-497. PMID:10215732

**附件一 知情同意书**

**知情同意书**

版本号：1.1

患者姓名缩写：□□□□

随机号：□□□

研究中心：□□

研究科室：▁▁▁▁▁

研究者：▁▁▁▁▁

监查员：▁▁▁▁▁

2013年9月

**尊敬的受试者**

医生已经确诊您为幽门螺杆菌感染相关性胃炎，我们邀请您参加“三联疗法联合聚普瑞锌（3+P疗法）治疗幽门螺杆菌相关性胃炎”多中心、随机、平行对照临床研究，本研究方案已得到 协和医院药物临床试验伦理委员会审核同意进行临床研究。在您决定是否参加这项研究之前，请您仔细阅读以下内容，它可以帮助您了解该项研究以及为何要进行这项研究，研究的程序和期限，参加研究后可能给您带来的受益和风险。如果您愿意，您可以和您的亲属，朋友一起讨论，也可以向您的医生询问，以确定您理解有关内容。

1. **研究背景和研究目的**

**1.1 疾病负担和治疗现况**

幽门螺杆菌（Hp）感染是慢性胃炎的重要病因，根除Hp是治疗慢性胃炎和预防复发的重要手段。Hp感染的治疗是目前研究领域的重点和热点课题，Hp根除治疗方案主要包括三联和四联疗法。随着时间变迁，长时间和频繁应用抗生素出现Hp对抗生素耐药现象，导致Hp根除率越来越低，目前阿莫西林和克拉霉素是临床中较为常用的两个抗生素。如何提高Hp根除率，如何选择更有效的治疗方案即合理的用药组合是当前临床医生普遍关注的问题。

聚普瑞锌颗粒（商品名：瑞莱生）是唯一一个L-肌肽与锌的螯合剂，研究发现，其具有多途径阻断幽门螺杆菌的独特药理作用，即通过阻断幽门螺杆菌定植、抑制其活性、清除尿素酶及一氯胺、抑制炎症反应达到提高幽门螺杆菌根除率的目的。

国外进行了聚普瑞锌与兰索拉唑，克拉霉素及阿莫西林三联疗法联合应用7天的幽门螺杆菌根治情况的研究。本研究共收集66名因感染幽门螺杆菌而消化不良的患者，将其随机分配到两种治疗方法组，一组（LAC；31例）给予兰索拉唑30毫克，每日两次；阿莫西林500毫克，每日两次，克拉霉素400毫克，每日两次，共7天。另一组（LACP；35例）在第一组给药方法的基础上加用聚普瑞锌150毫克，每日两次，共7天。在给药开始及4周后通过快速尿素试验、组织学和培养的方法评估幽门螺杆菌感染状态。结果显示，5名患者没有完成治疗，其中LAC组中的2名患者没有进行后续内窥镜检查；LAC组中的1名和LACP组中的2名患者由于严重腹泻终止治疗。根据完成治疗分析法，在LAC治疗后，28名可评估的患者中有24名达到幽门螺杆菌根治，（占86%，95%可信区间：72-100%）；在LACP组中，33名可评估患者有33例幽门螺杆菌达到根治（100%）（P<0.05）。根据意向治疗分析法，LAC组中的根治率为31例患者中24例根治（77%，95%可信区间：62-93%），而LACP组的为35例患者33例根治（94%，95%可信区间：86-100%），差异具有统计学意义（P<0.05）。说明兰索拉唑，克拉霉素及阿莫西林三联疗法在联合应用聚瑞普锌后根治幽门螺杆菌效果明显增强。其幽门螺杆菌根除率从77%提升到94%，具有显著差异性。

随着生活质量的提高，生活节奏的加快，Hp阳性的胃炎患者逐年增多，严重者可发展为溃疡甚至胃癌，严重影响了患者的生活质量甚至危及患者生命，因而，应及时采取有效的手段进行治疗。目前治疗Hp阳性胃炎的常用方法为质子泵抑制剂联合两种抗生素，但近年抗生素耐药性明显增加，Hp根除率有所下降。

由吉林省博大伟业制药有限公司研制的聚普瑞锌颗粒，属化学药品第3.1类新药，于2012年在中国上市。本着严谨、科学循证的态度，在本品上市后将对其进行深入研究，验证其提高Hp根除率的有效性。目前尚未发现服用该药有严重不良反应的报导，国外本药品说明书上显示少数患者发生有肝功能损伤、皮疹、瘙痒、荨麻疹、嗜酸性细胞增多，白血球减少，血小板减少，便秘、嗳气、腹胀、呕吐、胸闷、腹泻等。

**1.2研究目的**

评估和比较不同剂量聚普瑞锌联合三联疗法较三联疗法提高幽门螺杆菌根除率的临床有效性和安全性。

1.3 研究参加单位和预计纳入参试者例数

本次试验由北京协和医院为临床试验负责单位，上海仁济医院、南昌大学附属第一医院、中南大学湘雅医院、武汉大学人民医院、山东大学齐鲁医院、郑州医科大学第一附属医院、哈尔滨医科大学附属第一医院、厦门大学附属中山医院、吉林大学第一临床医院、南京医科大学第一附属医院为参加单位，对其进行多中心临床研究。拟入组共330例患者，每个分中心30例患者。

1. **哪些人不宜参加研究**

（1）年龄<18岁或>70岁; （2）既往Hp根除治疗史；（3）半年内胃镜存在胃溃疡、胃癌等胃部重大疾病；（4）青霉素皮试阳性；（5）治疗前4周应用抗生素、铋剂史；（6）治疗前2周应用PPI史；（7）合并严重肝病、心脏病、肾病、恶性肿瘤及酒精中毒等严重疾病；（8）精神病、严重神经官能症，不能合作本试验者；（9）对本研究中药物过敏；（10）妊娠或哺乳期妇女；（11）入组前3个月内参加其它临床试验

**三、研究内容和步骤**

在接受治疗中，如果发现诊断为合并Hp感染的胃炎患者，且临床特征符合本实验入组条件，研究医生将建议您在以后的治疗中参与本实验。在治疗周期过程中，您将分别得到免费提供本品和三联药品的治疗，试验期间研究医生将严密观察您的治疗情况，让您得到有效的医学治疗和服务。

本研究为多中心、随机、平行对照试验设计。330例患者按1:1:1比例随机分入治疗组a（奥美拉唑、阿莫西林、克拉霉素联合聚普瑞锌颗粒75mg）、治疗组b（奥美拉唑、阿莫西林、克拉霉素联合聚普瑞锌颗粒150mg）或对照组（奥美拉唑、阿莫西林、克拉霉素），分别服用试验药或对照药。具体步骤如下：

1.入组前筛选

如果您在治疗过程中诊断为合并Hp的胃炎，同意参加本研究并签署本知情同意书，入组前七天内您将免费接收一下项目检查，以确定您是否适合参加本项研究。

| 病史 | 生命体征 | 心电图 | C13/C14尿素呼气试验 |
| --- | --- | --- | --- |
| 肾功能 | 血常规 | 尿常规 | 肝功能 |
| 尿妊娠检查（生育期妇女） | |  | |

如果您经过检查，符合入选标准，您将被随机分入治疗组a、治疗组b或对照组接受相应治疗。

2.试验中的治疗与检查

本研究为连续治疗2周，连续治疗2周后停药。

在治疗前医生将做相关检查以决定您是否能够参加该项研究。在接受药物治疗的期间，要求您每周定期来医院接受相应的检查。并将您所填写的患者日记卡交由您的主管医生检查，同时领取您下一周期服用的药物。你必须将在研究期间自行服用的其他药物，以及您打算在该项研究期间使用的药物和疑问告知您的医生。这一点非常重要，如果您不便按照上述要求或者为了您的安全，医生也可能让您退出该项研究，接受其他替代治疗。特别提醒的是，如果您是妊娠妇女，则不宜参加本项研究。育龄妇女应该在试验期间采取有效避孕措施。

**四、参加研究可能的受益**

本项研究将为您免费提供试验所需的药品和与试验本身相关的化验及检查费用，如果没有特殊情况请您完成本项研究的全过程。您还将得到更好的Hp根除效果，使您的临床症状得以改善，虽然很多临床试验已经确认该药的临床疗效，但是我们无法保证每一个受试者都会在该项研究中得到满意疗效。

**五、参加研究可能的不良反应、风险和不适、不方便**

任何药物都有可能发生不良反应。目前尚未发现服用聚普瑞锌有严重不良反应的报导，国外本药品说明书上显示少数患者发生有肝功能损伤、皮疹、瘙痒、荨麻疹、便秘、嗳气、腹胀、呕吐、胸闷、腹泻等，医生会严密观察您的病情变化并及时处理，研究过程中的任何改变和任何新近明确的不良反应都会如实地告诉您。

对于Hp阳性的胃炎患者，临床主要的治疗方法为质子泵抑制剂联合两种抗生素，本研究中所应用的质子泵抑制剂为阿斯利康制药有限公司生产的奥美拉唑，所选用的抗生素分别为西安利君制药有限责任公司生产的克拉霉素及联邦制药生产的阿莫西林。

在服用奥美拉唑期间可能出现头痛和腹泻、恶心、便秘等胃肠道症状，其发生率在1～3%。

在克拉霉素的临床研究中，发现成人最常见的不良反应是胃肠不适，如恶心、消化不良、腹痛呕吐和腹泻，其它不良反应包括头痛、味觉异常和肝转氨酶短暂升高。很少有报道称，克拉霉素造成肝功能异常，如肝转氨酶升高、黄疸或无黄疸的肝细胞性和/或胆汁淤积性（药物性）肝炎，这种异常可能会很严重但经常是可逆转的。极少数有肝坏死的报道，且多与严重疾病和/或同服其它药物有关，罕见胰腺炎和惊厥报告。极少病例引起血清肌酐浓度升高，但原因不明。口服克拉霉素，曾有发生过敏反应报告，轻者为荨麻疹和轻度皮疹，重者为过敏和Stevens-Johnson氏综合症或毒性表皮坏死。曾有发生短暂性中枢神经系统的不良反应报告，包括头昏、眩晕、焦虑、失眠、噩梦、耳鸣、意识模糊、定向力障碍、幻觉和精神病，但因果关系不清楚。有报道表明，克拉霉素会导致可逆转的失聪、味觉改变和味觉失调。克拉霉素治疗期间，会发生舌炎、胃炎、口腔念球菌和舌无色。也有牙变色的报告，但牙变色可通过专业牙科逆转。极少有发生低血糖症的报告，有些是同服降血糖药或胰岛素造成。极少会发生白细胞和血小板减少症。与其他大环内酯类药物类似，克拉霉素很少会导致Q-T间期延长，心律失常如室性心动过速，室颤和充血性心力衰竭，免疫低下患者。对AIDS和其他免疫低下的病人采用较高剂量的克拉霉素长期治疗分支杆菌感染时，很难区分副作用究竟是克拉霉素造成还是HIV病症或并发症。对成年患者，克拉霉素每天总剂量为1克时的最常见不良反应是：恶心、呕吐、味觉失调、腹泻、皮疹、胃气胀、头痛、便秘、听觉障碍、SGOT和SGPT升高，白细胞和血小板数减少，较少患者还出现BUN升高。

在服用阿莫西林期间，可能发生的不良反应为恶心、呕吐、腹泻及假膜性肠炎等胃肠道反应；皮疹、药物热和哮喘等过敏反应；贫血、血小板减少、嗜酸性粒细胞增多等；血清氨基转移酶可轻度增高；由念珠菌或耐药菌引起的二重感染；偶见兴奋、焦虑、失眠、头晕以及行为异常等中枢神经系统症状。

任何药物均可能产生一定的不良反应，故不能保证本品一定有效，也不能排除发生不良反应的可能。但在试验中发现任何可能影响您健康的情况，研究医生会及时告知您或您的合法代表，并积极采取有效的治疗措施，保护您的合法权益。

**六、研究相关费用**

在您参与本研究期间，您所使用的试验药物（本品和奥美拉唑、阿莫西林、克拉霉素药品）由申办单位免费提供，本研究方案规定的C13/C14尿素呼气试验、血常规、尿常规、肾功能、肝功能、心电图、尿妊娠检查检查，亦由申办单位支付相关检查费用。但是，与本项研究无关的费用，则由您或您的医疗保险机构负担。如果您对费用有疑问，可以咨询您的研究医生。在研究期间，如果您发生与本研究药物相关的不良事件或严重不良事件时，研究医生会采取积极的治疗措施，并且治疗产生的相关费用均由承办方承担。如果在临床试验中出现不良事件，医学专家委员会将会鉴定其是否治疗药物有关。申办者将按照我国《药物临床试验质量管理规范》的规定对与试验相关的损害提供治疗的费用及相应的经济补偿。对于您同时合并的其他疾病所需的治疗和检查，将不在免费的范围之内。

**七、医疗记录的公开及保密**

在有关法律规定下。您的医疗记录将被保密。但研究人员、监查人员、伦理委员会成员或药政管理人员可能查阅您的医疗记录。如果研究结果公开发表，您的身份不会被公开。签署本文件表示您已经授权，研究人员可以使用上述您的医疗记录。

您同意在本研究中获得的资料（包括您已进行的所有检查结果），保存在研究者的计算机和书面文档系统中，但实验资料中不会出现您的姓名。您也同意将这些试验资料用于在我国或其它国家药品注册的目的，在此过程中申办单位将使用这些记录，并按有关法律法规规定予以保存和保密。

**八、遵从的义务**

在整个研究期间，您必须遵守本品研究方案的有关规定，有义务配合医护人员，详细记录试验相关的信息资料，保证研究资料的真实性。如果您未经负责医生的同意使用其他药物，或有妨碍试验完成的行为，研究人员可要求您退出试验，或提前终止试验。

**九、自愿参加/退出研究**

参与本研究以自愿为原则，可拒绝参与或在任何时候均可退出，您将不会因此受到歧视或失去任何权益，更不会受到任何不公正的待遇。如果希望退出研究，您必须通知研究医生。当然，如果继续参加本研究对您不利，研究医生会在任何时候，甚至未征得您同意时就可终止您参与本研究。伦理委员会及申办单位也有可能终止本研究。

如果提前退出研究，医生将要求您做最后一次检查和评估。在相关法律/法规允许范围内，退出之前已获得的研究资料仍可能会被采用。

**知情同意书**

**患者**（或代理人）**姓名：**

- 我在此确定医生已向我告知了聚普瑞锌颗粒临床研究的目的、方法、获益和可能的风险。
- 我已经阅读并理解了关于受试者资讯的说明。
- 我已经知道研究的结果将在临床研究总结报告中进行分析，但个人资料如：姓名、生日和诊断结果等会受到保密。
- 我可以在任何时候退出该项研究。
- 我有足够的机会提出问题并表明我是否自愿参加该项研究。
- 我已被告知口服该药可能发生不良反应，如在试验过程中发生与试验有关的不良反应研究单位和申办者将给予积极的治疗，并依据国家有关规定，您将得到相应的补偿。
- 我同意在试验期间采取适当的避孕措施（育龄妇女）。
- 我已知道本项研究已经得到本医院伦理委员会讨论批准。

本知情同意书一式两份，请受试者和研究中心妥善保管，以备药品注册审评部门审查。

**患者**（或代理人）**：我已阅读并完全理解上述的有关医学研究的资料，我确认已有充足的时间考虑，有关疑问已经得到圆满解答，我自愿同意参加本临床研究。**

患者（或代理人）姓名： 与患者关系：

日期： 年 月 日

**联系电话：1、手机： 2、宅电：**

家庭住址：

**主管医生：我确认已向患者解释了本项研究的详细情况，包括其权利及可能的获益和风险；并且保证随时解答患者关于本项临床研究信息的咨询。**

医生签名： 日期： 年 月 日

**联系电话：**

**附件二 原始病例表**

**三联疗法联合聚普瑞锌（3+P疗法）治疗幽门螺杆菌相关性胃炎多中心、随机、平行对照临床研究**

**原始病例表**

**版本号：1.0**

**日期：2013年9月11日**

**受试者姓名缩写：□□□□**

**随 机 号：□□□**

**中 心 编 号：□□**

**研 究 科 室：**

**研 究 者：**

**监 察 员：**

**患者基本信息**

患者姓名 性别

年龄 民族

家庭住址

联系电话

**入组前筛选**

**知情同意书**

签署日期： 年 月 日

**一般资料**

受试者姓名缩写： 病案号：

性别: 男 女 民族： 汉族 其他

出生日期： 年 月 日 年龄： 岁

身高： cm 体重： . kg 体表面积： . m² 身高： cm 体重： . kg 体表面积： . m²

身高： cm 体重： . kg 体表面积： . m²

**C13尿素呼气试验**

阳性，≥4.0±0.4

阴性，＜4.0±0.4

**C14 尿素呼气试验**

阳性，^14^C-UBT≥100dpm/mmolCO_2_

阴性，^14^C-UBT＜100dpm/mmolCO_2_

**既往史**

**无 有，请填写下表**

| **疾病**  **序号** | **部位/**  **系统代码** | **疾病名称** | **诊断时间**  **（年/月）** | **是否治愈** |
| --- | --- | --- | --- | --- |
| **1** |  |  |  |  |
| **2** |  |  |  |  |
| **3** |  |  |  |  |
| **4** |  |  |  |  |

**部位/系统代码**

01=呼吸系统 02=心血管系统 03=胃肠道系统

04=肝脏 05=内分泌/代谢 06=中枢神经系统

07=血液/淋巴系统 08=皮肤 09=肌肉骨骼

10=生殖泌尿系统 11=精神系统 12=酒精/药物滥用

13=药物过敏 14=非药物过敏 93=其他

**生命体征**

检查日期： 年 月 日

体温： ℃ 血压： / mmHg

心率： 次/分 呼吸： 次/分

**体格检查**

检查日期： 年 月 日

体格检查是否有异常？

否 是，在下表记录异常

身体系统代码 异常状况描述


身体系统代码：

01=头眼耳鼻喉 02=皮肤 03=淋巴结 04=胸部/肺 05=心脏/心血管

06=腹部 07=上肢 08=下肢 09=神经系统 93=其他

**血常规**

检查日期： 年 月 日

| 指标 | 结果 | 单位 | 临床意义判定 | | | | | |
| --- | --- | --- | --- | --- | --- | --- | --- | --- |
|  |  |  | 正常 | 异常，无临床意义 | | 异常，有临床意义 | 未查 | |
| 血小板计数 |  | 10^9^/L |  | |  |  | |  |
| 白细胞计数 |  | 10^9^/L |  | |  |  | |  |
| 淋巴细胞计数 |  | 10^9^/L |  | |  |  | |  |
| 中性粒细胞计数 |  | 10^9^/L |  | |  |  | |  |
| 红细胞计数 |  | 10^12^/L |  | |  |  | |  |
| 血红蛋白 |  | g/L |  | |  |  | |  |
| 红细胞压积 |  | **-** |  | |  |  | |  |

**尿常规**

检查日期： 年 月 日

| 指标 | 结果 | 临床意义判定 | | | |
| --- | --- | --- | --- | --- | --- |
|  |  | 正常 | 异常，无临床意义 | 异常，有临床意义 | 未查 |
| PH值 |  |  |  |  |  |
| 红细胞 |  |  |  |  |  |
| 白细胞 |  |  |  |  |  |
| 尿胆原 |  |  |  |  |  |
| 尿蛋白 | □阴性□阳性 |  |  |  |  |
| 尿糖 | □阴性□阳性 |  |  |  |  |
| 尿胆红素 | □阴性□阳性 |  |  |  |  |
| 尿酮体 | □阴性□阳性 |  |  |  |  |
| 尿亚硝酸盐 | □阴性□阳性 |  |  |  |  |

**肝功能**

检查日期： 年 月 日

| 指标 | 结果 | 单位 | 临床意义判定 | | | |
| --- | --- | --- | --- | --- | --- | --- |
|  |  |  | 正常 | 异常，无临床意义 | 异常，有临床意义 | 未查 |
| 谷丙转氨酶 |  | U/L | □ | □ | □ | □ |
| 谷草转氨酶 |  | U/L | □ | □ | □ | □ |
| 总胆红素 |  | μmol/L | □ | □ | □ | □ |
| 直接胆红素 |  | μmol/L | □ | □ | □ | □ |
| 总蛋白 |  | g/L | □ | □ | □ | □ |
| 白蛋白 |  | g/L | □ | □ | □ | □ |
| 血糖 |  | mmol/L | □ | □ | □ | □ |

**肾功能**

检查日期： 年 月 日

**EKG**

| 指标 | 结果 | 单位 | 临床意义判定 | | | |
| --- | --- | --- | --- | --- | --- | --- |
|  |  |  | 正常 | 异常，无临床意义 | 异常，有临床意义 | 未查 |
| 尿素氮 |  | mmol/L | □ | □ | □ | □ |
| 肌酐 |  | μmol/L | □ | □ | □ | □ |

检查日期： 年 月 日

结果判定：□正常

□异常，无临床意义

□异常，有临床意义，请描述

□未查

**入选标准判断**

| 下述各项均为是，才能进入筛选周期 | 是 | 否 |
| --- | --- | --- |
| 1.半年内有过胃镜检查诊断为胃炎，并排除有胃溃疡、胃癌等胃部重大疾病患者。 |  |  |
| 2.C13/C14尿素呼气试验Hp阳性者。 |  |  |
| 3.年龄18～70岁，男女不限。 |  |  |
| 4.Hp阳性初治患者。 |  |  |
| 5.青霉素皮试无过敏者。 |  |  |
| 6.签署知情同意书者 |  |  |

**排除标准判断**

| 下述各项均为否，才能进入筛选周期 | 是 | 否 |
| --- | --- | --- |
| 1.治疗前4周用过抗生素、铋剂或者治疗前2周用过PPI者。 |  |  |
| 2.妊娠或哺乳期妇女。 |  |  |
| 3.病人同时存在其它影响本研究评价的严重疾病如严重的肝病、心脏病、肾脏病、恶性肿瘤及酒精中毒疾患。 |  |  |
| 4.入组前3个月内参加过其他临床试验者。 |  |  |
| 5.对本研究所用药物过敏者，或既往有青霉素过敏史者。 |  |  |
| 6.病人不能正确表达自己主诉，如精神病、严重神经官能症，不能合作本试验者。 |  |  |

**入组判定**

判定日期： 年 月 日

是否符合所有标准

□是 □否（在下表中填写不符合的入组或排除标准）

不符合标准 标准编号 不符合标准 标准编号

入选标准 入选标准

排除标准 排除标准

是否有入选/排除标准豁免 □是，请填写下表 □否

豁免解释：

豁免确认签字：

□符合标准，入组

□符合所有标准，但患者撤销知情同意书

□不符合标准，退出

**随机分组**

随机号：

□治疗组:

剂量：每日两次，聚普瑞锌75mg/次,洛赛克20mg/次，阿莫西林1000mg/次,克拉霉素：500mg/次。

□治疗组:

剂量:每日两次，聚普瑞锌150mg/次,洛赛克20mg/次，阿莫西林1000mg/次,克拉霉素：500mg/次。

□对照组:

剂量:洛赛克20mg/次，阿莫西林1000mg/次,克拉霉素：500mg/次。

**用药记录**

**受试药物使用记录**

| 用量： mg | |
| --- | --- |
| 给药日期 | 年 月 日至 年 月 日 |

**试验流程**

**聚普瑞锌颗粒临床研究流程表**

| 项 目 | 治疗前 | 治疗过程 | | 疗程结束后 |
| --- | --- | --- | --- | --- |
|  | 0周（-5～-1天） | 1周末 | 2周末 | 6-8周内 |
| 筛 选 | × |  |  |  |
| 签署知情同意书 | × |  |  |  |
| 确定纳入排除标准 | × |  |  |  |
| 病史采集 | × |  |  |  |
| 生命体征 | × | × | × | × |
| 随机分组 | × |  |  |  |
| 血常规检查 | × |  |  | × |
| 尿常规检查 | × |  |  | × |
| 肾功能检查 | × |  |  | × |
| 肝功能检查 | × |  |  | × |
| 妊娠检查（生育期妇女） | × |  |  |  |
| 心电图 | × |  |  | × |
| C13/C14尿素呼气试验 | × |  |  | × |
| 治疗与随访 |  | × | × | × |
| 合并用药 | × | × | × | × |
| 记录不良反应 |  | × | × | × |
| 回收剩余药品 |  |  | × |  |

注: “×”为必须按随访时间检查的项目或操作内容。

**疗程结束4周末检查：**

**生命体征**

检查日期： 年 月 日

体温： ℃ 血压： / mmHg

心率： 次/分 呼吸： 次/分

**血常规**

**检查日期： 年 月 日**

| 指标 | 结果 | 单位 | 临床意义判定 | | | | | |
| --- | --- | --- | --- | --- | --- | --- | --- | --- |
|  |  |  | 正常 | 异常，无临床意义 | | 异常，有临床意义 | 未查 | |
| 血小板计数 |  | 10^9^/L |  | |  |  | |  |
| 白细胞计数 |  | 10^9^/L |  | |  |  | |  |
| 淋巴细胞计数 |  | 10^9^/L |  | |  |  | |  |
| 中性粒细胞计数 |  | 10^9^/L |  | |  |  | |  |
| 红细胞计数 |  | 10^12^/L |  | |  |  | |  |
| 血红蛋白 |  | g/L |  | |  |  | |  |
| 红细胞压积 |  | **-** |  | |  |  | |  |

**尿常规**

检查日期： 年 月 日

| 指标 | 结果 | 临床意义判定 | | | |
| --- | --- | --- | --- | --- | --- |
|  |  | 正常 | 异常，无临床意义 | 异常，有临床意义 | 未查 |
| PH值 |  |  |  |  |  |
| 红细胞 |  |  |  |  |  |
| 白细胞 |  |  |  |  |  |
| 尿胆原 |  |  |  |  |  |
| 尿蛋白 | □阴性□阳性 |  |  |  |  |
| 尿糖 | □阴性□阳性 |  |  |  |  |
| 尿胆红素 | □阴性□阳性 |  |  |  |  |
| 尿酮体 | □阴性□阳性 |  |  |  |  |
| 尿亚硝酸盐 | □阴性□阳性 |  |  |  |  |

**肝功能**

检查日期： 年 月 日

| 指标 | 结果 | 单位 | 临床意义判定 | | | |
| --- | --- | --- | --- | --- | --- | --- |
|  |  |  | 正常 | 异常，无临床意义 | 异常，有临床意义 | 未查 |
| 谷丙转氨酶 |  | U/L | □ | □ | □ | □ |
| 谷草转氨酶 |  | U/L | □ | □ | □ | □ |
| 总胆红素 |  | μmol/L | □ | □ | □ | □ |
| 直接胆红素 |  | μmol/L | □ | □ | □ | □ |
| 总蛋白 |  | g/L | □ | □ | □ | □ |
| 白蛋白 |  | g/L | □ | □ | □ | □ |
| 血糖 |  | mmol/L | □ | □ | □ | □ |

**肾功能**

检查日期： 年 月 日

| 指标 | 结果 | 单位 | 临床意义判定 | | | |
| --- | --- | --- | --- | --- | --- | --- |
|  |  |  | 正常 | 异常，无临床意义 | 异常，有临床意义 | 未查 |
| 尿素氮 |  | mmol/L | □ | □ | □ | □ |
| 肌酐 |  | μmol/L | □ | □ | □ | □ |

**EKG**

检查日期： 年 月 日

结果判定：□正常

□异常，无临床意义

□异常，有临床意义，请描述

□未查

**C13尿素呼气试验**

阳性，≥4.0±0.4

阴性，＜4.0±0.4

**C14 尿素呼气试验**

阳性，^14^C-UBT≥100dpm/mmolCO_2_

阴性，^14^C-UBT＜100dpm/mmolCO_2_

**合并用药**

| 药物通用名称 | 用药原因 | 每次用量 | 给药途径 | 用法 | 用药日期 |
| --- | --- | --- | --- | --- | --- |
|  |  |  | □□ | □□ |  |
|  |  |  | □□ | □□ |  |
|  |  |  | □□ | □□ |  |
|  |  |  | □□ | □□ |  |
|  |  |  | □□ | □□ |  |
|  |  |  | □□ | □□ |  |

给药途径编号 用法编号

01=口服 01=每日1次 09=每4小时1次

02=静脉注射 02=每日2次 10=每6小时1次

03=肌肉注射 03=每日3次 11=每8小时1次

04=皮下注射 04=每日4次 12=每12小时1次

05=局部给药 05=每隔1日 13=连续滴注

06=鞘内给药 06=每周1次 14=根据需要

93=其他 07=每周2次 15=未知

08=每周3次 93=其他

**不良事件**

| （用标准医学术语）记录所有观察到的和用以下问句“自上次检查后，您有何不同的感觉？”直接询问得出的不良事件。尽量使用诊断名称而不使用症状名称。每一栏记录一个不良事件。  **该受试者整个试验期间有无经历任何不良事件？ □有 □无 如“有”请分别填下表。** | | | | |
| --- | --- | --- | --- | --- |
| **不良事件名称**  （填写字迹要清晰） |  |  | |  |
| **开始发生日期和时间** | _______年＿＿月＿＿日  ＿＿＿：＿＿（24小时制） | _______年＿＿月＿＿日  ＿＿＿：＿＿（24小时制） | | _______年＿＿月＿＿日  ＿＿＿：＿＿（24小时制） |
| **不良事件严重程度** | □轻 □中 □重 | □轻 □中 □重 | | □轻 □中 □重 |
| **是否采取措施**  （如是，请记录伴随用药和伴随治疗记录表） | □是 □否 | □是 □否 | | □是 □否 |
| **对研究药物**  **的剂量影响** | □ 剂量不变 □ 增加剂量  □ 减小剂量 □ 暂停用药  □ 永久停药 | □ 剂量不变 □ 增加剂量  □ 减小剂量 □ 暂停用药  □ 永久停药 | | □ 剂量不变 □ 增加剂量  □ 减小剂量 □ 暂停用药  □ 永久停药 |
| **与研究药物的关系** | □ 肯定有关 □ 可能有关  □ 可能无关 □ 无关  □ 无法判定 | □ 肯定有关 □ 可能有关  □ 可能无关 □ 无关  □ 无法判定 | | □ 肯定有关 □ 可能有关  □ 可能无关 □ 无关  □ 无法判定 |
| **根据研究者的判断是否符合严重不良事件定义？**  1.导致死亡.  2.威胁生命.  3.导致住院或延长住院期间.  4.导致持续或严重残疾/能力丧失.  5.导致先天性异常或出生缺陷.  6.重要医学事件（如有可能影响到受试者并有可能需要药物/手术以防止上述结果） | □ 是 □ 否  （如是，请立即电话/传真报告0431-81158782和0431-881158731）  报告日期： ＿＿年＿月＿日 | □ 是 □ 否  （如是，请立即电话/传真报告0431-81158732和0431-881158731）  报告日期： ＿＿年＿月＿日 | | □ 是 □ 否  （如是，请立即电话/传真报告0431-81158732和0431-81158731）  报告日期： ＿＿年＿月＿日 |
| **在不良事件终止或研究结束时填写以下部分** | | | | |
| **所发生不良**  **事件的结局** | □ 仍存在 □ 已缓解  □ 不知道  缓解日期： ＿＿年＿月＿日 | | □ 仍存在 □ 已缓解  □ 不知道  缓解日期： ＿＿年＿月＿日 | □ 仍存在 □ 已缓解  □ 不知道  缓解日期： ＿＿年＿月＿日 |
| **患者是否因此不良**  **事件而退出试验？** | □ 是 □ 否 | | □ 是 □ 否 | □ 是 □ 否 |

**严重不良反应**

严重不良事件报告表（**SAE**）

| 报告类型 | □首次报告□ 随访报告 □总结报告 | | | | | 报告时间： 年 月 日 | | | | |
| --- | --- | --- | --- | --- | --- | --- | --- | --- | --- | --- |
| 医疗机构及专业名称 |  | | | | | 电话 | |  | | |
| 申办单位名称 |  | | | | | 电话 |  | | | |
| 试验用药品名称 | 中文名称： | | | | | | | | | |
|  | 英文名称 | | | | | | | | | |
| 药品类别 | □中药 □化学药 □ 新生物制品 □放射性药 □进口药 □其它 | | | | | | | | 第 类 | |
| 临床研究分期 | □Ⅰ期 □Ⅱ期 □Ⅲ 期 □Ⅳ期 □生物等效性试验 □临床验证 | | | | | | | | 剂型: | |
| 受试者情况 | 姓名： | | 性别： | | 出生时间： | | | | | 民族： |
|  | 疾病诊断： | | | | | | | | | |
| SAE情况 | □导致住院 □ 延长住院时间 □伤残 □功能障碍  □导致先天畸形 □危及生命或死亡 □其它 | | | | | | | | | |
| SAE发生时间： 年 月 日 | | | | SAE反应严重程度: □ 轻度 □中度 □重度 | | | | | | |
| 对试验用药采取的措施 | | □继续用药 □ 减小剂量 □ 药物暂停后又恢复 □停用药物 | | | | | | | | |
| SAE转归 | | □症状消失(后遗症：□ 有 □无)  □症状持续□死亡(死亡时间： 年 月 日) | | | | | | | | |
| SAE与试验药的关系 | | □肯定 □很可能 □ 可能 □可疑 □ 不可能 □未评价 □无法评价 | | | | | | | | |
| 破盲情况 | | □未破盲  □已破盲（破盲时间： 年 月 日） | | | | | | | | |
| SAE报道情况 | | 国内： □有 □无 □不详  国外： □有 □无 □不详 | | | | | | | | |
| SAE发生及处理的详细情况: | | | | | | | | | | |

**附件三 病例报告表（CRF）**

**三联疗法联合聚普瑞锌（3+P疗法）治疗幽门螺杆菌相关性胃炎多中心、随机、平行对照临床研究**

**病例报告表（CRF）**

**版本号：1.0**

**日期：2013年9月11日**

**受试者姓名缩写：□□□□**

**随 机 号：□□**

**中 心 编 号：□□**

**研 究 科 室：**

**研 究 者：**

**监 察 员：**

**病例报告表填写说明**

**在正式填表前，请认真阅读以下内容**

1.筛选合格者填写正式病例报告表。

2.病例报告表由三联复写纸组成，一式三份，需用蓝黑或黑色签字笔用力填写。

3.填写数据及内容务必准确、清晰，不得随意涂改。如发现填写内容有误，纠正错误时需用横线居中划出，在旁边写明正确内容，同时签署修改者姓名缩写及修改日期。

4.受试者姓名缩写需填满四格。两字姓名填写两字拼音的前两个字母；三字姓名填写三字首字母再加第三字的第二字母；四字姓名填写每个字的首字母。

例如：张红ZHHO；李书明LSMI；欧阳小惠OYXH

5.对于选择项目，需在相应项目的方框内用╳标注；对于表格中所有栏目，需填写相应的数字或文字，在填写阿拉伯数字时，缺省方框用0填写。如因故未查或漏查，则此项填入“ND”；如药物使用情况不明，则此项填入“NK”；如不能提供或不适用，则此项填入“NA”；多余表格划“/”。

6.试验期间要如实填写不良事件记录表。需记录不良事件的名称、发生时间、严重程度、持续时间、采取的措施和转归。如有严重不良事件发生，必须立即通知本院伦理委员会，组长单位伦理委员会及申办单位。

**入组前筛选**

**知情同意书**

签署日期： 年 月 日

**一般资料**

受试者姓名缩写： 病案号：

性别: 男 女 民族： 汉族 其他

出生日期： 年 月 日 年龄： 岁

身高： cm 体重： . kg 体表面积： . m² 身高： cm 体重： . kg 体表面积： . m²

身高： cm 体重： . kg 体表面积： . m²

**C13尿素呼气试验**

阳性，≥4.0±0.4

阴性，＜4.0±0.4

**C14 尿素呼气试验**

阳性，^14^C-UBT≥100dpm/mmolCO_2_

阴性，^14^C-UBT＜100dpm/mmolCO_2_

**既往史**

无 有，请填写下表

| **疾病**  **序号** | **部位/**  **系统代码** | **疾病名称** | **诊断时间**  **（年/月）** | **是否治愈** |
| --- | --- | --- | --- | --- |
| **1** |  |  |  |  |
| **2** |  |  |  |  |
| **3** |  |  |  |  |
| **4** |  |  |  |  |

**部位/系统代码**

01=呼吸系统 02=心血管系统 03=胃肠道系统

04=肝脏 05=内分泌/代谢 06=中枢神经系统

07=血液/淋巴系统 08=皮肤 09=肌肉骨骼

10=生殖泌尿系统 11=精神系统 12=酒精/药物滥用

13=药物过敏 14=非药物过敏 93=其他

**生命体征**

检查日期： 年 月 日

体温： ℃ 血压： / mmHg

心率： 次/分 呼吸： 次/分

**体格检查**

检查日期： 年 月 日

体格检查是否有异常？

否 是，在下表记录异常

身体系统代码 异常状况描述


身体系统代码：

01=头眼耳鼻喉 02=皮肤 03=淋巴结 04=胸部/肺 05=心脏/心血管

06=腹部 07=上肢 08=下肢 09=神经系统 93=其他

**血常规**

检查日期： 年 月 日

| 指标 | 结果 | 单位 | 临床意义判定 | | | | | |
| --- | --- | --- | --- | --- | --- | --- | --- | --- |
|  |  |  | 正常 | 异常，无临床意义 | | 异常，有临床意义 | 未查 | |
| 血小板计数 |  | 10^9^/L |  | |  |  | |  |
| 白细胞计数 |  | 10^9^/L |  | |  |  | |  |
| 淋巴细胞计数 |  | 10^9^/L |  | |  |  | |  |
| 中性粒细胞计数 |  | 10^9^/L |  | |  |  | |  |
| 红细胞计数 |  | 10^12^/L |  | |  |  | |  |
| 血红蛋白 |  | g/L |  | |  |  | |  |
| 红细胞压积 |  | **-** |  | |  |  | |  |

**尿常规**

检查日期： 年 月 日

| 指标 | 结果 | 临床意义判定 | | | |
| --- | --- | --- | --- | --- | --- |
|  |  | 正常 | 异常，无临床意义 | 异常，有临床意义 | 未查 |
| PH值 |  |  |  |  |  |
| 红细胞 |  |  |  |  |  |
| 白细胞 |  |  |  |  |  |
| 尿胆原 |  |  |  |  |  |
| 尿蛋白 | □阴性□阳性 |  |  |  |  |
| 尿糖 | □阴性□阳性 |  |  |  |  |
| 尿胆红素 | □阴性□阳性 |  |  |  |  |
| 尿酮体 | □阴性□阳性 |  |  |  |  |
| 尿亚硝酸盐 | □阴性□阳性 |  |  |  |  |

**肝功能**

检查日期： 年 月 日

| 指标 | 结果 | 单位 | 临床意义判定 | | | |
| --- | --- | --- | --- | --- | --- | --- |
|  |  |  | 正常 | 异常，无临床意义 | 异常，有临床意义 | 未查 |
| 谷丙转氨酶 |  | U/L | □ | □ | □ | □ |
| 谷草转氨酶 |  | U/L | □ | □ | □ | □ |
| 总胆红素 |  | μmol/L | □ | □ | □ | □ |
| 直接胆红素 |  | μmol/L | □ | □ | □ | □ |
| 总蛋白 |  | g/L | □ | □ | □ | □ |
| 白蛋白 |  | g/L | □ | □ | □ | □ |

**肾功能**

检查日期： 年 月 日

| 指标 | 结果 | 单位 | 临床意义判定 | | | |
| --- | --- | --- | --- | --- | --- | --- |
|  |  |  | 正常 | 异常，无临床意义 | 异常，有临床意义 | 未查 |
| 尿素氮 |  | mmol/L | □ | □ | □ | □ |
| 肌酐 |  | μmol/L | □ | □ | □ | □ |
| 尿酸 |  | μmol/L | □ | □ | □ | □ |

**EKG**

检查日期： 年 月 日

结果判定：□正常

□异常，无临床意义

□异常，有临床意义，请描述

□未查

**尿妊娠检查**

是否进行尿妊娠检查 □否 □是

如否，请描述原因：□受试者已无生育能力

□其它，请描述

如是，请填写下述信息：

检查日期： 年 月 日

结果： □阴性 □阳性

入选标准判断

| 下述各项均为是，才能进入筛选周期 | 是 | 否 |
| --- | --- | --- |
| 1.半年内有过胃镜检查，曾排除有胃溃疡、胃癌等胃部重大疾病患者。 |  |  |
| 2.C13/C14尿素呼气试验阳性者。 |  |  |
| 3.年龄18～70岁，男女不限。 |  |  |
| 4. Hp阳性初治患者。 |  |  |
| 5.青霉素皮试无过敏者。 |  |  |
| 6.签署知情同意书患者。 |  |  |

排除标准判断

| 下述各项均为否，才能进入筛选周期 | 是 | 否 |
| --- | --- | --- |
| 1.治疗前4周用过抗生素、铋剂或者治疗前2周用过PPI者。 |  |  |
| 2.妊娠或哺乳期妇女。 |  |  |
| 3.病人同时存在其它影响本研究评价的严重疾病如严重的肝病、心脏病、肾脏病、恶性肿瘤及酒精中毒疾患。 |  |  |
| 4.入组前3个月内参加过其他临床试验者。 |  |  |
| 5.对本研究所用药物过敏者，或既往有青霉素过敏史者。 |  |  |
| 6.病人不能正确表达自己主诉，如精神病、严重神经官能症，不能合作本试验者。 |  |  |

**入组判定**

判定日期： 年 月 日

是否符合所有标准

□是 □否（在下表中填写不符合的入组或排除标准）

不符合标准 标准编号 不符合标准 标准编号

入选标准 入选标准

排除标准 排除标准

是否有入选/排除标准豁免 □是，请填写下表 □否

豁免解释：

豁免确认签字：

□符合标准，入组

□符合所有标准，但患者撤销知情同意书

□不符合标准，退出

**随机分组**

随机号：

□治疗组:

剂量：每日两次，聚普瑞锌75mg/次，洛赛克20mg/次，阿莫西林1000mg/次，克拉霉素500mg/次。

□治疗组:

剂量：每日两次，聚普瑞锌150mg/次，洛赛克20mg/次，阿莫西林1000mg/次，克拉霉素500mg/次。

□对照组:

剂量：洛赛克20mg/次，阿莫西林1000mg/次，克拉霉素500mg/次。

**用药记录**

**受试药物使用记录**

| 用量： mg | |
| --- | --- |
| 给药日期 | 年 月 日至 年 月 日 |

**试验流程**

聚普瑞锌颗粒临床研究流程表

| 项 目 | 治疗前 | 治疗过程 | | 疗程结束后 |
| --- | --- | --- | --- | --- |
|  | 0周（-5～-1天） | 1周末 | 2周末 | 6-8周内 |
| 筛 选 | × |  |  |  |
| 签署知情同意书 | × |  |  |  |
| 确定纳入排除标准 | × |  |  |  |
| 病史采集 | × |  |  |  |
| 生命体征 | × | × | × | × |
| 随机分组 | × |  |  |  |
| 血常规检查 | × |  |  | × |
| 尿常规检查 | × |  |  | × |
| 肾功能检查 | × |  |  | × |
| 肝功能检查 | × |  |  | × |
| 妊娠检查（生育期妇女） | × |  |  |  |
| 心电图 | × |  |  | × |
| C13/C14尿素呼气试验 | × |  |  | × |
| 治疗与随访 |  | × | × | × |
| 合并用药 | × | × | × | × |
| 记录不良反应 |  | × | × | × |
| 回收剩余药品 |  |  | × |  |

注: “×”为必须按随访时间检查的项目或操作内容。

**疗程结束4周末检查：**

**生命体征**

检查日期： 年 月 日

体温： ℃ 血压： / mmHg

心率： 次/分 呼吸： 次/分

**血常规**

检查日期： 年 月 日

| 指标 | 结果 | 单位 | 临床意义判定 | | | | | |
| --- | --- | --- | --- | --- | --- | --- | --- | --- |
|  |  |  | 正常 | 异常，无临床意义 | | 异常，有临床意义 | 未查 | |
| 血小板计数 |  | 10^9^/L |  | |  |  | |  |
| 白细胞计数 |  | 10^9^/L |  | |  |  | |  |
| 淋巴细胞计数 |  | 10^9^/L |  | |  |  | |  |
| 中性粒细胞计数 |  | 10^9^/L |  | |  |  | |  |
| 红细胞计数 |  | 10^12^/L |  | |  |  | |  |
| 血红蛋白 |  | g/L |  | |  |  | |  |
| 红细胞压积 |  | **-** |  | |  |  | |  |

**尿常规**

检查日期： 年 月 日

| 指标 | 结果 | 临床意义判定 | | | |
| --- | --- | --- | --- | --- | --- |
|  |  | 正常 | 异常，无临床意义 | 异常，有临床意义 | 未查 |
| PH值 |  |  |  |  |  |
| 红细胞 |  |  |  |  |  |
| 白细胞 |  |  |  |  |  |
| 尿胆原 |  |  |  |  |  |
| 尿蛋白 | □阴性□阳性 |  |  |  |  |
| 尿糖 | □阴性□阳性 |  |  |  |  |
| 尿胆红素 | □阴性□阳性 |  |  |  |  |
| 尿酮体 | □阴性□阳性 |  |  |  |  |
| 尿亚硝酸盐 | □阴性□阳性 |  |  |  |  |

**肝功能**

检查日期： 年 月 日

| 指标 | 结果 | 单位 | 临床意义判定 | | | |
| --- | --- | --- | --- | --- | --- | --- |
|  |  |  | 正常 | 异常，无临床意义 | 异常，有临床意义 | 未查 |
| 谷丙转氨酶 |  | U/L | □ | □ | □ | □ |
| 谷草转氨酶 |  | U/L | □ | □ | □ | □ |
| 总胆红素 |  | μmol/L | □ | □ | □ | □ |
| 直接胆红素 |  | μmol/L | □ | □ | □ | □ |
| 总蛋白 |  | g/L | □ | □ | □ | □ |
| 白蛋白 |  | g/L | □ | □ | □ | □ |

**肾功能**

检查日期： 年 月 日

| 指标 | 结果 | 单位 | 临床意义判定 | | | |
| --- | --- | --- | --- | --- | --- | --- |
|  |  |  | 正常 | 异常，无临床意义 | 异常，有临床意义 | 未查 |
| 尿素氮 |  | mmol/L | □ | □ | □ | □ |
| 肌酐 |  | μmol/L | □ | □ | □ | □ |
| 尿酸 |  | μmol/L | □ | □ | □ | □ |

**EKG**

检查日期： 年 月 日

结果判定：□正常

□异常，无临床意义

□异常，有临床意义，请描述

□未查

**C13尿素呼气试验**

阳性，≥4.0±0.4

阴性，＜4.0±0.4

**C14 尿素呼气试验**

阳性，^14^C-UBT≥100dpm/mmolCO_2_

阴性，^14^C-UBT＜100dpm/mmolCO_2_

合并用药

| 药物通用名称 | 用药原因 | 每次用量 | 给药途径 | 用法 | 用药日期 |
| --- | --- | --- | --- | --- | --- |
|  |  |  | □□ | □□ |  |
|  |  |  | □□ | □□ |  |
|  |  |  | □□ | □□ |  |
|  |  |  | □□ | □□ |  |
|  |  |  | □□ | □□ |  |
|  |  |  | □□ | □□ |  |

给药途径编号 用法编号

01=口服 01=每日1次 09=每4小时1次

02=静脉注射 02=每日2次 10=每6小时1次

03=肌肉注射 03=每日3次 11=每8小时1次

04=皮下注射 04=每日4次 12=每12小时1次

05=局部给药 05=每隔1日 13=连续滴注

06=鞘内给药 06=每周1次 14=根据需要

93=其他 07=每周2次 15=未知

08=每周3次 93=其他

不良事件

| （用标准医学术语）记录所有观察到的和用以下问句“自上次检查后，您有何不同的感觉？”直接询问得出的不良事件。尽量使用诊断名称而不使用症状名称。每一栏记录一个不良事件。  **该受试者整个试验期间有无经历任何不良事件？ □有 □无 如“有”请分别填下表。** | | | | |
| --- | --- | --- | --- | --- |
| **不良事件名称**  （填写字迹要清晰） |  |  | |  |
| **开始发生日期和时间** | _______年＿＿月＿＿日  ＿＿＿：＿＿（24小时制） | _______年＿＿月＿＿日  ＿＿＿：＿＿（24小时制） | | _______年＿＿月＿＿日  ＿＿＿：＿＿（24小时制） |
| **不良事件严重程度** | □轻 □中 □重 | □轻 □中 □重 | | □轻 □中 □重 |
| **是否采取措施**  （如是，请记录伴随用药和伴随治疗记录表） | □是 □否 | □是 □否 | | □是 □否 |
| **对研究药物**  **的剂量影响** | □ 剂量不变 □ 增加剂量  □ 减小剂量 □ 暂停用药  □ 永久停药 | □ 剂量不变 □ 增加剂量  □ 减小剂量 □ 暂停用药  □ 永久停药 | | □ 剂量不变 □ 增加剂量  □ 减小剂量 □ 暂停用药  □ 永久停药 |
| **与研究药物的关系** | □ 肯定有关 □ 可能有关  □ 可能无关 □ 无关  □ 无法判定 | □ 肯定有关 □ 可能有关  □ 可能无关 □ 无关  □ 无法判定 | | □ 肯定有关 □ 可能有关  □ 可能无关 □ 无关  □ 无法判定 |
| **根据研究者的判断是否符合严重不良事件定义？**  1.导致死亡.  2.威胁生命.  3.导致住院或延长住院期间.  4.导致持续或严重残疾/能力丧失.  5.导致先天性异常或出生缺陷.  6.重要医学事件（如有可能影响到受试者并有可能需要药物/手术以防止上述结果） | □ 是 □ 否  （如是，请立即电话/传真报告0431-81158732/0431-881158731）  报告日期： ＿＿年＿月＿日 | □ 是 □ 否  （如是，请立即电话/传真报告0431-81158732/0431-881158731）  报告日期： ＿＿年＿月＿日 | | □ 是 □ 否  （如是，请立即电话/传真报告0431-81158732/0431-81158731）  报告日期： ＿＿年＿月＿日 |
| **在不良事件终止或研究结束时填写以下部分** | | | | |
| **所发生不良**  **事件的结局** | □ 仍存在 □ 已缓解  □ 不知道  缓解日期： ＿＿年＿月＿日 | | □ 仍存在 □ 已缓解  □ 不知道  缓解日期： ＿＿年＿月＿日 | □ 仍存在 □ 已缓解  □ 不知道  缓解日期： ＿＿年＿月＿日 |
| **患者是否因此不良**  **事件而退出试验？** | □ 是 □ 否 | | □ 是 □ 否 | □ 是 □ 否 |

**严重不良反应**

严重不良事件报告表（**SAE**）

| 报告类型 | □首次报告□ 随访报告 □总结报告 | | | | | 报告时间： 年 月 日 | | | | |
| --- | --- | --- | --- | --- | --- | --- | --- | --- | --- | --- |
| 医疗机构及专业名称 |  | | | | | 电话 | |  | | |
| 申办单位名称 |  | | | | | 电话 |  | | | |
| 试验用药品名称 | 中文名称： | | | | | | | | | |
|  | 英文名称 | | | | | | | | | |
| 药品类别 | □中药 □化学药 □ 新生物制品 □放射性药 □进口药 □其它 | | | | | | | | 第 类 | |
| 临床研究分期 | □Ⅰ期 □Ⅱ期 □Ⅲ 期 □Ⅳ期 □生物等效性试验 □临床验证 | | | | | | | | 剂型: | |
| 受试者情况 | 姓名： | | 性别： | | 出生时间： | | | | | 民族： |
|  | 疾病诊断： | | | | | | | | | |
| SAE情况 | □导致住院 □ 延长住院时间 □伤残 □功能障碍  □导致先天畸形 □危及生命或死亡 □其它 | | | | | | | | | |
| SAE发生时间： 年 月 日 | | | | SAE反应严重程度: □ 轻度 □中度 □重度 | | | | | | |
| 对试验用药采取的措施 | | □继续用药 □ 减小剂量 □ 药物暂停后又恢复 □停用药物 | | | | | | | | |
| SAE转归 | | □症状消失(后遗症：□ 有 □无)  □症状持续□死亡(死亡时间： 年 月 日) | | | | | | | | |
| SAE与试验药的关系 | | □肯定 □很可能 □ 可能 □可疑 □ 不可能 □未评价 □无法评价 | | | | | | | | |
| 破盲情况 | | □未破盲  □已破盲（破盲时间： 年 月 日） | | | | | | | | |
| SAE报道情况 | | 国内： □有 □无 □不详  国外： □有 □无 □不详 | | | | | | | | |
| SAE发生及处理的详细情况: | | | | | | | | | | |

**试验结束总结表**

□ 按照试验方案要求完成，试验正常结束

□ 试验中途终止，请填写下列各项

终止时，试验所处阶段： □ 第一周

□ 第二周

□ 试验最后一次检查

中途终止的原因：

□ 不良事件

□ 受试者要求退出研究

□ 违背试验方案

□ 因治疗需要改变治疗方案

□ 主要研究者认为退出研究有利于受试者

□ 申办方决定

□ 其他

**声 明**

我已经仔细阅读该患者的CRF表格的全部内容，并保证根据我本人的判断表格中所填写的内容是真实而准确，并符合研究方案要求。

研 究 者 签 名： 日期： 年 月 日

研究负责人签名： 日期： 年 月 日

检 查 员 签 名： 日期： 年 月 日
