# Supplementary material for: Polaprezinc combined with clarithromycin-based triple therapy for Helicobacter pylori-associated gastritis: A prospective, multicenter, randomized clinical trial
Source: PLoS One. 2017 Apr 13;12(4):e0175625. doi: 10.1371/journal.pone.0175625 (PMC5391070; doi:10.1371/journal.pone.0175625)
Supplement: S3 File — (DOCX) [file pone.0175625.s003.docx]

**Polaprezinc Combined with Clarithromycin-Based Triple Therapy for *Helicobacter pylori*–Associated Gastritis: A Prospective, Multicenter, Randomized Clinical Trial**

| **Research Center** | **Principal Investigator** |
| --- | --- |
| Peking Union Medical College Hospital | Prof. Jia-Ming Qian* |
| Renji Hospital Shanghai Jiaotong University School of Medicine | Prof. Jing-Yuan Fang |
| First Affiliated Hospital of Zhejiang University | Prof. Feng Ji |
| First Affiliated Hospital of Zhengzhou University | Prof. Jian-Sheng Li |
| Qilu Hospital of Shandong University | Prof. Yan-Qing Li |
| People's Hospital of Wuhan University | Prof. He-Sheng Luo |
| First Affiliated Hospital of Nanchang University | Prof. Nong-Hua Lv |
| Zhongshan Hospital of Xiamen University | Prof. Jian-Lin Ren |
| Jiangsu People's Hospital | Prof. Rui-Hua Shi |
| First Clinical Hospital of Jilin University | Prof. Hong Xu |
| Xiangya Hospital of Central South University | Prof. Yi-You Zou |

*****Chief Investigator


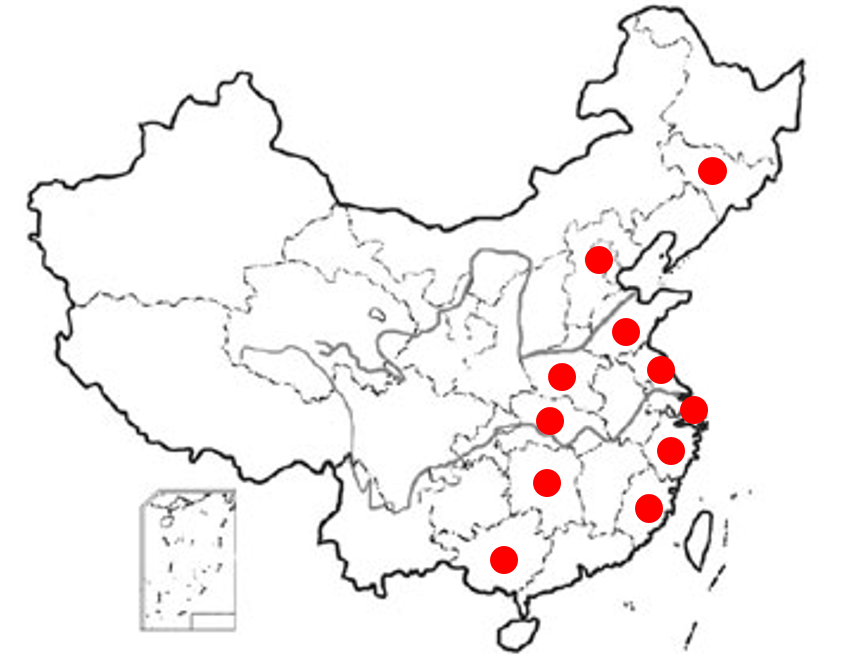


**Financial support:** This study was partially sponsored by Broadwell Pharmaceutical.

**Abstract**

This was a randomized, parallel-group controlled, prospective multicenter study conducted in 11 cities in China. Treatment-naive patients with *Helicobacter pylori*–associated gastritis were randomly assigned into either Experimental Group A triple therapy (omeprazole 20 mg, amoxicillin 1 g, and clarithromycin 500 mg twice daily) plus polaprezinc 75 mg twice daily for 14 days, Experimental Group B triple therapy plus polaprezinc 150 mg twice daily for 14 days, or the Control Group triple-therapy for 14 days. The primary endpoint was rate of *H. pylori* eradication. Symptom improvement and a lower incidence of adverse events were the secondary endpoints.

**Specific aims**

- Assess the clinical efficacy and safety of polaprezinc combined with triple therapy in the eradication of *H. pylori*
- Comparison the clinical efficacy of different doses of polaprezinc combined with triple therapy in the eradication of *H. pylori*
- Recommend an optimal clinical treatment to eradicate *H. pylori*

**Background**

*Helicobacter pylori*, one of the most prevalent global pathogens, colonizes an estimated 50% of the world’s population [1]. In China, the rate of *H. pylori* infection remains elevated at 40%–60%, in part due to increasing rates of antibiotic resistance and decreased rates of efficacy with proton pump inhibitor (PPI)–based triple therapy regimens [2]. Among the antibiotics recommended for *H. pylori* eradication therapy, the rate of resistance to metronidazole is as high as 60%–70%; to clarithromycin, 20%–38%; and to levofloxacin 30%–38%. Amoxicillin, furazolidone, and tetracycline resistance rates remain low at 1%–5%. With increased resistance to antibiotics, *H. pylori* eradication rates using standard triple therapy (i.e., PPI + clarithromycin + amoxicillin) have dropped below 80%. Even when therapy is extended from 7 days to 10 days, the eradication rate increases only by about 5%. Sequential therapy, concomitant therapy, and levofloxacin-based triple therapy have not demonstrated any advantage in native Chinese patients [2]. Bismuth-containing quadruple therapy is currently the recommended front-line treatment in China. However, some patients cannot tolerate bismuth (e.g., allergy) due to the gastrointestinal symptoms they experience, such as nausea, vomiting, and darkened stools. Also, the ideal regimen for bismuth (i.e., compound, formulation, dose, dosing interval, and relation to meals) remain unclear [3]. The most recent Chinese *H. pylori* infection consensus report suggests that mucosal protective agents combined with triple therapy may have the same efficacy as bismuth-containing quadruple therapy [4]. However, there have been very few clinical studies evaluating the efficacy of adding mucosal protective agents to enhance eradication therapy for *H. pylori* in China.

Polaprezinc is a gastric mucosal protective drug complex of zinc and l-carnosine with a more than 20-year history of clinical use in Japan. In addition to its role as a mucosal protective agent that promotes the healing of peptic ulcers, it also improves *H. pylori* eradication rates [5, 6]. In 1999, Kashimura and colleagues found that polaprezinc combined with a triple therapy comprising lansoprazole, amoxicillin, clarithromycin can increase the *H. pylori* eradication rate from 24/31 (77.4%) with triple therapy alone to 33/35 (94.3%), with no increase in the incidence of adverse events [5]. Based on this single-center, small sample size study in Japan, we designed a randomized, parallel-group controlled, open-label, prospective multicenter study to evaluate the clinical efficacy and safety of adding polaprezinc to triple therapy to improve the rate of *H. pylori* eradication in Chinese patients with gastritis. We also compared the clinical efficacy of two different doses polaprezinc combined with triple therapy in the eradication of *H. pylori* and improvement of clinical symptoms*.*

**Methods**

**Overview of design: We conducted** a randomized, parallel-controlled, open-label prospective multicenter clinical study.


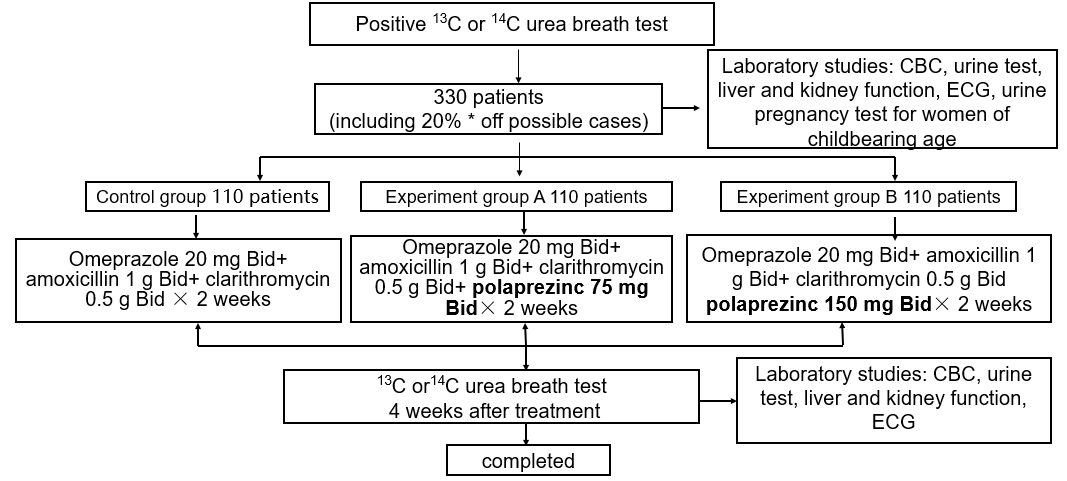


**Clinical Trial Flow Chart**

| Items | Baseline | Treatment process | | After treatment |
| --- | --- | --- | --- | --- |
|  | 0 week | 1 week | 2 weeks | 6–8 weeks |
| Screening | × |  |  |  |
| Obtain signed informed consent | × |  |  |  |
| Evaluate inclusion & exclusion criteria | × |  |  |  |
| Collect medical history | × |  |  |  |
| Measure vital signs | × | × | × | × |
| Randomize | × |  |  |  |
| Complete blood count | × |  |  | × |
| Urine test | × |  |  | × |
| Kidney function | × |  |  | × |
| Liver function | × |  |  | × |
| Urine pregnancy test | × |  |  |  |
| Electrocardiogram | × |  |  | × |
| ^13^C or ^14^C urea breath test | × |  |  | × |
| Treatment and follow-up |  | × | × | × |
| Combination drugs | × | × | × | × |
| Incidence of adverse events |  | × | × | × |
| Recover drugs |  |  | × |  |

**Study Subjects**

**Participants:** Adult patients with *H. pylori*–related gastritis who visited 11 participating hospitals from January 2014. The hospitals included Peking Union Medical College Hospital, Chinese Academy of Medical Science & Peking Union Medical College; First Clinical Hospital of Jilin University; First Affiliated Hospital of Nanchang University; Jiangsu People’s Hospital; People's Hospital of Wuhan University; First Affiliated Hospital of Zhengzhou University; Zhongshan Hospital of Xiamen University; Xiangya Hospital of Central South University; Qilu Hospital of Shandong University; First Affiliated Hospital of Zhejiang University; and Renji Hospital Shanghai Jiaotong University School of Medicine.

**Selection Criteria**

**Inclusion criteria:**

- Age 18–70 years
- ^13^C or ^14^C urea breath test positive for first-time therapy
- Complete gastroscopy to exclude ulcer and gastric cancer within 6 months
- Penicillin skin test positive
- Signed informed consent

**Exclusion criteria:**

- Taking antibiotics or bismuth 4 weeks prior to therapy
- Taking PPI 2 weeks prior to therapy
- Patients with severe liver, heart, kidney disease, alcoholism, malignancy, or other serious diseases
- Psychosis, severe neurosis
- Allergy to polaprezinc or penicillin
- pregnant or lactating women
- Participated in other clinical trials within 3 months prior to therapy

**Measurements**

**Interventions**

- All enrolled participants underwent a physical examination and laboratory examinations, including blood and routine urine test, measurement of liver and kidney functions, ECG, urine pregnancy test for women of childbearing age.
- Then, participants were randomly divided into one of three groups: control group, Experimental Group A, or Experimental Group B.
- Control Group: omeprazole 20 mg + amoxicillin 1 g + clarithromycin 0.5 g orally twice daily for 14 days.
- Experimental Group A: polaprezinc 75 mg+ omeprazole 20 mg + amoxicillin 1 g + clarithromycin 0.5 g orally twice daily for 14 days.
- Experimental Group B: polaprezinc 150 mg+ omeprazole 20 mg + amoxicillin 1 g + clarithromycin 0.5 g orally twice daily for 14 days.
- All patients underwent the ^13^C or ^14^C urea breath test 4 weeks after the completion of the intervention, as well as the physical examination and laboratory studies.

**Drugs**

- Polaprezinc (Ruilaisheng^®^, Broadwell Pharmaceutical, Jilin, China)
- Omeprazole (Losec^®^, AstraZeneca Pharmaceutical, Shanghai, China)
- Amoxicillin (Federal Amoxil, Zhuhai Federal Pharmaceutical, Zhuhai, China)
- Clarithromycin (Limaixian^®^, Xi’an Lijun Pharmaceutical, Xi’an, China)

**Endpoint Variables**

**Primary endpoint variables:** The primary endpoint of this study was *H. pylori* eradication 4 weeks after the completion of the intervention, as measured by a ^13^C or ^14^C urea breath test.

- ^13^C urea breath test (≥ 4.0 ± 0.4) or ^14^C urea breath test (^14^C-UBT ≥ 100 dpm/mmol CO_2_) were regarded as positive, and the participant was defined as *H. pylori* infected

**Secondary endpoint variables:** The secondary endpoints were the improvement of digestive symptoms by symptom day 7, 14, and 28 after completion of intervention, and a lower incidence of adverse effects.

- Measured symptoms included upper abdominal pain, acid reflux, belching, heartburn, bloating, nausea, and vomiting, which were each divided to 4 categories of severity: 0 presenting none, 1 presenting mild, 2 presenting moderate, and 3 presenting severe.
- Enrolled participants were asked to record these symptoms before treatment and on day 7, 14, and 28 after treatment. The patients were informed of the common adverse effects before treatment and were asked to record these symptoms during treatment. Follow-up interviews were arranged for day 7, 14, and 28 after treatment.

**Sample size estimates**

The sample size was calculated based on the primary endpoint. A previous single-center, small sample size study conducted in Japan reported that polaprezinc combined with triple therapy increased the *H. pylori* eradication rate from 77.4% to 94.3% [5]. With a two-sided α value of 0.05 and β value of 0.20, we estimated that we would need a sample size of 112 patients in each group to detect a 15% difference.

**Randomization and Blinding**

**Randomization:** The randomization schedule for each center was generated by the Department of Epidemiology and Health Statistics, Peking Union Medical College. When an eligible participant was ready to be randomized, the investigator provided the name and study ID of the new participant to an independent researcher who randomized the participant into Arm A, Arm B, or Arm C, according to a predetermined random list supplied to each site. This independent researcher also hamdles distribution and recovery of the drugs.

**Blinding:** This trial was designed as open-lable, for the primary endpoint eradication rate of *H. pylori* is the objective results.

**Adverse Events**

**Definition:** Any adverse medical event reported by the participant or the physician should be recorded from the time when the participant signed the informed consent form up to and including the last follow-up, whether or not the adverse event is related to the study drug. Abnormal changes in the physical examination or the laboratory studies after treatment should be recorded as an adverse event.

**Record:** The occurrence of adverse events, the relationship to the study drug, severity, duration, treatment taken, and outcomes should be faithfully recorded during the study. Adverse events should be recorded on the case report form (CRF) Adverse Event Report Form.

**Severity:** When completing the CRF Adverse Event Report Form, the investigator should use mild, moderate, and severe to characterize the adverse event. For a unified standard, the severity of adverse events was graded as follows:

- Mild: Does not affect the normal function of the subject
- Moderate: To some extent affects the normal function of subject
- Severe: Significantly affects the normal function of the subject

**Relationship:** The investigator should evaluate the possible relationship between adverse events and the study drug or the combination of drugs, and determine if there is a causal relationship between the adverse event and the study drug based on the following criteria:

1. Whether there is a reasonable relationship between the time point of medication initiation and the time point of adverse event occurrence
2. Whether the suspected adverse event is consistent with the type of adverse reaction known to associated with the drug
3. Whether the suspected adverse event can be explained by the effect of the combination of drugs, the clinical status of the patient, or the effects of other therapies
4. Whether withdrawal or reduced dose of study drug causes the adverse event to disappear or to be less severe.
5. Whether the same reaction occurs if the study drug is taken again.

According to the above principles, relationship is characterized as follows: definitely related, probably related, possibly related, possibly unrelated, and unrelated.

|  | 1 | 2 | 3 | 4 | 5 |
| --- | --- | --- | --- | --- | --- |
| Definitely related | + | + | - | + | + |
| Probably related | + | + | - | + | ? |
| Possibly related | + | + | ± | ± | ? |
| Possibly unrelated | + | - | ± | ± | ? |
| Unrelated | - | - | + | - | - |

+ yes; – no; ± not sure; ? unknown

**Severe Adverse Events**

**Definition:** An adverse event would be classified as a serious adverse event (SAE) if one or more of the following criteria were met:

- Death
- Life-threatening (e.g., risk of immediate death)
- Condition leads to hospitalization or hospitalization of prolonged duration
- Permanent or severe disability
- Congenital malformations or defects
- The SAE should be considered as a medical event that has not resulted in death, life-threatening or hospitalization. The SAE should also be considered by the investigating physician as potentially harmful to the patient and requiring treatment by medication or surgery to avoid the occurrence of above conditions.

**Treatment:** The patient should be withdrawn and given the appropriate treatment.

**Record and report:** Any serious adverse event in the course of clinical research must be reported to the investigator and to the Peking Union Medical College Hospital Clinical Research Center, and also reported to the Ethics Committee. The investigator must complete a Serious Adverse Event Reporting Form, which will record the time, severity, duration, treatment taken, and outcome of the serious adverse event.

If the patient suddenly dies during the test period, in addition to the Serious Adverse Event Report, an autopsy report should be completed if the patient receives an autopsy.

**Statistical Issues**

**Analysis plan:** All statistical analyses were performed with SAS 9.3 software (SAS Institute Irvine, California, CA) by the Department of Epidemiology and Health Statistics, Peking Union Medical College. The primary endpoint – *H. pylori* eradication rate – and one of the secondary endpoints – improvement of digestive symptoms – were analyzed with an intention-to-treat (ITT) population and a per-protocol (PP) population. The secondary endpoint – incidence of adverse events – was analyzed with a safety analysis set (SAS). The ITT population included all eligible patients who were enrolled and randomized, and who took at least one dose of drug. Missing observations were accounted for using the last observation carried forward (LOCF) method. The PP population included all eligible patients who completed the entire protocol with good compliance. The SAS population included all patients who were randomized and who took drugs and were assessed for safety at least once. Categorical variables are described as percentages, while continuous variables are described as means and standard deviations. Chi-square and ANOVA test were used for categorical variables and continuous variables analyzing respectively. The Cochran–Mantel–Haenszel test (CMH)–χ^2^ test was used for group comparisons, and the Mantel–Haenszel method was used to calculate the 95% confidence interval of the *H. pylori* eradication rate for each group; and the differences in eradication rates between 2 groups. All *P* values were two-tailed and *P* < 0.05 was considered statistically significant.

**Participant Management**

Participants would withdraw from the study if any of the following conditions occurred: (1) disease causing deterioration or serious complications; (2) serious adverse event; (3) participant had other disease that interfered with observation during treatment; (4) lost to follow-up; (5) pregnancy during treatment.

**Specimen and Drug Management**

Blood and urine tests were performed in the clinical laboratory of the respective hospitals, according to standard specimen and clinical testing processes.

Storage, distribution, and recovery all drugs was handled by an independent researcher.

**Data Management**

The data were collected, input, and saved by independent investigators according to the CRF. The data were sent to the Department of Epidemiology and Health Statistics of Peking Union Medical College for statistical analyses.

**Management of Posttrial Treatment**

The participants who failed *H. pylori* eradication were followed up, and retreated with *H. pylori* eradication therapy after 2–3 months, with the participant’s consent.

**References**

1. [Thung I](https://www.ncbi.nlm.nih.gov/pubmed/?term=Thung%20I%5BAuthor%5D&cauthor=true&cauthor_uid=26694080), [Aramin H](https://www.ncbi.nlm.nih.gov/pubmed/?term=Aramin%20H%5BAuthor%5D&cauthor=true&cauthor_uid=26694080), [Vavinskaya V](https://www.ncbi.nlm.nih.gov/pubmed/?term=Vavinskaya%20V%5BAuthor%5D&cauthor=true&cauthor_uid=26694080), [Gupta S](https://www.ncbi.nlm.nih.gov/pubmed/?term=Gupta%20S%5BAuthor%5D&cauthor=true&cauthor_uid=26694080), [Park JY](https://www.ncbi.nlm.nih.gov/pubmed/?term=Park%20JY%5BAuthor%5D&cauthor=true&cauthor_uid=26694080), [Crowe SE](https://www.ncbi.nlm.nih.gov/pubmed/?term=Crowe%20SE%5BAuthor%5D&cauthor=true&cauthor_uid=26694080), et al. Review article: the global emergence of *Helicobacter pylori* antibiotic resistance. [Aliment Pharmacol Ther](https://www.ncbi.nlm.nih.gov/pubmed/26694080). 2016;43:514-533.
2. Chinese Society of Gastroenterology, Chinese Study Group on Helicobacter pylori, Liu WZ, Xie Y, Cheng H, Lu NH, Hu FL, et al. Fourth Chinese National Consensus Report on the management of Helicobacter pylori infection. J Dig Dis. 2013;14:211-221.
3. [Dore MP](https://www.ncbi.nlm.nih.gov/pubmed/?term=Dore%20MP%5BAuthor%5D&cauthor=true&cauthor_uid=26848181), [Lu H](https://www.ncbi.nlm.nih.gov/pubmed/?term=Lu%20H%5BAuthor%5D&cauthor=true&cauthor_uid=26848181), [Graham DY](https://www.ncbi.nlm.nih.gov/pubmed/?term=Graham%20DY%5BAuthor%5D&cauthor=true&cauthor_uid=26848181). Role of bismuth in improving *Helicobacter pylori* eradication with triple therapy. [Gut.](https://www.ncbi.nlm.nih.gov/pubmed/26848181) 2016; 65:870-878.
4. [Liang J](https://www.ncbi.nlm.nih.gov/pubmed/?term=Liang%20J%5BAuthor%5D&cauthor=true&cauthor_uid=23066868), [Li J](https://www.ncbi.nlm.nih.gov/pubmed/?term=Li%20J%5BAuthor%5D&cauthor=true&cauthor_uid=23066868), [Han Y](https://www.ncbi.nlm.nih.gov/pubmed/?term=Han%20Y%5BAuthor%5D&cauthor=true&cauthor_uid=23066868), [Xia J](https://www.ncbi.nlm.nih.gov/pubmed/?term=Xia%20J%5BAuthor%5D&cauthor=true&cauthor_uid=23066868), [Yang Y](https://www.ncbi.nlm.nih.gov/pubmed/?term=Yang%20Y%5BAuthor%5D&cauthor=true&cauthor_uid=23066868), [Li W](https://www.ncbi.nlm.nih.gov/pubmed/?term=Li%20W%5BAuthor%5D&cauthor=true&cauthor_uid=23066868), et al. *Helicobacter pylori* eradication with ecabet sodium, omeprazole, amoxicillin, and clarithromycin versus bismuth, omeprazole, amoxicillin, and clarithromycin quadruple therapy: a randomized, open-label, phase IV trial. [Helicobacter.](https://www.ncbi.nlm.nih.gov/pubmed/23066868) 2012;17:458-465.
5. [Kashimura H](https://www.ncbi.nlm.nih.gov/pubmed/?term=Kashimura%20H%5BAuthor%5D&cauthor=true&cauthor_uid=10215732), [Suzuki K](https://www.ncbi.nlm.nih.gov/pubmed/?term=Suzuki%20K%5BAuthor%5D&cauthor=true&cauthor_uid=10215732), [Hassan M](https://www.ncbi.nlm.nih.gov/pubmed/?term=Hassan%20M%5BAuthor%5D&cauthor=true&cauthor_uid=10215732), [Ikezawa K](https://www.ncbi.nlm.nih.gov/pubmed/?term=Ikezawa%20K%5BAuthor%5D&cauthor=true&cauthor_uid=10215732), [Sawahata T](https://www.ncbi.nlm.nih.gov/pubmed/?term=Sawahata%20T%5BAuthor%5D&cauthor=true&cauthor_uid=10215732), [Watanabe T](https://www.ncbi.nlm.nih.gov/pubmed/?term=Watanabe%20T%5BAuthor%5D&cauthor=true&cauthor_uid=10215732), et al. Polaprezinc, a mucosal protective agent, in combination with lansoprazole, amoxicillin and clarithromycin increases the cure rate of *Helicobacter pylori* infection. Aliment Pharmacol Ther. 1999;13:483-497.
6. [Sakae K](https://www.ncbi.nlm.nih.gov/pubmed/?term=Sakae%20K%5BAuthor%5D&cauthor=true&cauthor_uid=24691900), [Yanagisawa H](https://www.ncbi.nlm.nih.gov/pubmed/?term=Yanagisawa%20H%5BAuthor%5D&cauthor=true&cauthor_uid=24691900). Oral treatment of pressure ulcers with polaprezinc (zinc l-carnosine complex): 8-week open-label trial. [Biol Trace Elem Res.](https://www.ncbi.nlm.nih.gov/pubmed/?term=Oral+treatment+of+pressure+ulcers+with+polaprezinc+(zinc+L-carnosine+complex)%3A+8-week+open-label+trial) 2014;158:280-288.
