# Supplementary material for: Polaprezinc combined with clarithromycin-based triple therapy for Helicobacter pylori-associated gastritis: A prospective, multicenter, randomized clinical trial
Source: PLoS One. 2017 Apr 13;12(4):e0175625. doi: 10.1371/journal.pone.0175625 (PMC5391070; doi:10.1371/journal.pone.0175625)
Supplement: S4 File — (PDF) [file pone.0175625.s004.pdf]

13C906

北京协和医院药物临床试验伦理委员会会议审查批件

项目编号： 000923

药物/医疗器械名称	聚普瑞锌颗粒	注册分类	化学药品	申请事项	上市后申办者发起
申请人	吉林省博大伟业制药有限公司	任务来源	/		
专业组	消化内科	主要研究者	钱家鸣		
药审批件编号	无	药检/检验报告批号	25-130210,120301,BCCZ,1302089,30405006		
会议地点	北京协和医院临床药理研究中心会议室	会议日期	2013-09-11		

主要研究者资格评价：

有丰富的消化内科临床经验，现任科主任及中华消化协会副主任委员。曾经药物临床试验及GCP培训，任主要研究者接收完成多项药物临床试验。

研究方案名称：

三联疗法联合聚普瑞锌（3+P疗法）治疗幽门螺旋杆菌相关性胃炎多中心、随机、平行对照临床研究

研究方案编号：无 版本号：1.0

设计方案评价：

方案设计基本合理、可行。受试者安全有基本保障。

知情同意书评价：

基本符合要求。

版本号：：1.0

受试者补偿措施评价：

基本符合要求。

其他：

无

出席人数 8人 弃权人数 0人 回避人数 0人

投票结果	同意	8 票	审查决定	☑ 同意
	作必要的修正后同意	0 票		☐ 作必要的修正后同意
	作必要的修正后重审	0 票		☐ 作必要的修正后重审
	不同意	0 票		☐ 不同意
	终止或暂停已经批准的临床试验	0 票		☐终止或暂停已经批准的临床试验

跟踪审查频率为： ☐3个月 ☐6个月 ☑1年 ☐无 ☐其他

记录人签字：	白桦	主任委员/副主任委员签字：	翟晓梅

*本次审批的资料见附件。

北京协和医院药物临床试验伦理委员会

中国医学科学院北京协和医院 药物临床试验伦理委员会 1000000030774

会议签到表

会议日期：2013-9-11

会议地点：北京协和医院临床药理研究中心会议室

姓名	性别	出生年月	工作单位、职称	职务	签名
鲁重美	女	1949.8	北京协和医院消化内科，教授	主任委员	
翟晓梅	女	1956.12	中国医学科学院/中国协和医科大学生命伦理学研究中心，教授	副主任委员	翟晓梅
蔡金生	男	1943.6	中国全国工商业联合会，退休	委员	蔡金生
曹文莉	女	1967.5	北京市高默克律师事务所，律师	委员	曹文莉
单渊东	男	1941.1	北京协和医院血液内科，教授	委员	单渊东
游　凯	男	1933.3	北京协和医院心内科，教授	委员	
李大魁	男	1944.8	北京协和医院药剂科，教授	委员	
江　骥	男	1954.3	北京协和医院 I 期临床试验研究室，教授	委员	江骥
叶铁虎	男	1946.8	北京协和医院麻醉科，教授	委员	叶铁虎
崔丽英	女	1956.12	北京协和医院神经科，教授	委员	
张抒扬	女	1963.3	北京协和医院心内科，教授	委员	张抒扬
白　桦	女	1970.2	北京协和医院心内科，主治医师	委员	白桦

本伦理委员会是独立的，并遵守 ICH GCP、中国 GCP 及当地相关法规。所有出席的委员均在有效任职期间。本伦理委员会将对所审阅的临床研究资料以及伦理委员会会议讨论的结果和相关的内容保密，且与本研究项目无利益冲突。如果试验中发生任何严重不良事件请于 24 小时内通知本伦理委员会，并按相应跟踪审查频率及时上报临床试验情况。北京协和医院药物临床试验伦理委员会地址：北京西城区大木仓胡同 41 号。

联系人：孙燕。联系电话：010-69158355。特此声明。

北京协和医院药物临床试验伦理委员会

附件：

1. 新药证书
2. 药检报告
3. 专业组领导小组审批报告及主要研究者申请报告
4. 药品 GMP 证书和企业法人营业执照
5. 药品说明书
6. 试验方案
7. 知情同意书
8. CRF
9. 主要研究者、主要参与人员的简历及 GCP 培训证书复印件
10. 研究者会议纪要

北京协和医院药物临床试验伦理委员会
