# Supplementary material for: Polaprezinc combined with clarithromycin-based triple therapy for Helicobacter pylori-associated gastritis: A prospective, multicenter, randomized clinical trial
Source: PLoS One. 2017 Apr 13;12(4):e0175625. doi: 10.1371/journal.pone.0175625 (PMC5391070; doi:10.1371/journal.pone.0175625)
Supplement: S5 File — (PDF) [file pone.0175625.s005.pdf]

Clinical Research Ethical Approval by Peking Union Medical College Hospital Ethics Committee (Translation edition)

Project No.: 000923

Drug/Medical Apparatus	Polaprezinc	Registry Classification	Chemical medicine	Applic ation	Sponsor Launch After listing
Proposer	Jilin Province Broadwell Pharmaceutical Co.,Ltd	Project Source	N/A		
Department	Department of gastroenterology	Principle Investigators	Jiaming Qian		
Drug Review Approval Number	N/A	Batch Number of Inspection Report	25-130210, 120301, BCCZ, 1302089, 30405006		
Conference Location	Peking Union Medical College Hospital Ethics Committee Conference Room	Conference Date	2013-09-11		

Principal investigator qualifications assessment:

She has rich clinical experience of digestive diseases, director of gastroenterology departments of PUMCH and vice president of Chinese Society of Gastroenterology. She has received training of drug clinical trials and GCP. She has accepted and completed a number of drug clinical trials as principal investigator.

Name of research protocol:

Triple therapy combined with polaprezinc (3+P therapy) in treatment of *Helicobacter pylori* associated gastritis, a multicenter, randomized, parallel controlled clinical study.

Research project number: N/A Version No. 1.0

Designed protocol evaluation:

The protocol design is reasonable and feasible. The subjects have basic security.

Informed consent evaluation:

Satisfied with the requirements.

The subjects compensation measures evaluation:

Satisfied with the requirements.

Others:

N/A

Attendance: 8 **Waiver:** 0 **Avoid:** 0

Result of Voting	Agree:8 votes	Examined for Decision	☑ Agree
	Agree after revised:0 votes		☐Agree after revised
	Retrial after revised:0 votes		☐Retrial after revised
	Disagree:0 votes		☐Disagree
	End or pause the approved clinical trials:0 votes		☐ End or pause the approved clinical trials

Review follow up frequency: ☐3 month ☐6 month ☑1year ☐N/A ☐others

Recorder	Hua Bai	Chairman	Xiaomei Zhai

*Approval information on attachment

Peking Union Medical College Hospital Ethics Committee

Conference Attendance and Check-in Table

Conference Date：2013-9-11

Conference venue：Peking Union Medical College Hospital Ethics Committee Conference Room Conference Room

Name	Gender	Date of Birth	Work Unit, Title	Position	Signature
Chongmei Lu	Female	1949.8	Department of gastroenterology in Peking Union Medical College Hospital, Professor	Chairman	
Xiaomei Zhai	Female	1956.12	Chinese Academy of Medical Sciences/ Life Ethics Research Center in Peking Union Medical College Hospital, Professor	Vice Chairman	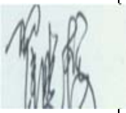
Jinsheng Cai	Male	1943.6	National Industry and Commerce Association, Retire	Committee	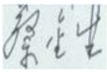
Wenli Cao	Female	1967.5	Beijing GMK Law Firm, Lawyer	Committee	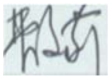
Yuandong Shan	Male	1941.1	Department of hematology in Peking Union Medical College Hospital, Professor	Committee	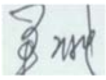
Kai You	Male	1933.3	Department of cardiology in Peking Union Medical College Hospital, Professor	Committee	
Dakui Li	Male	1944.8	Department of Pharmacy in Peking Union Medical College Hospital, Professor	Committee	
Ji Jiang	Male	1954.3	Phase I clinical study center of Peking Union Medical College Hospital, Professor	Committee	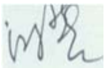
Tiehu Ye	Male	1946.8	Department of anesthesiology in Peking Union Medical College Hospital, Professor	Committee	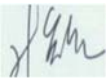
Liying Cui	Female	1956.12	Department of neurology in Peking Union Medical College Hospital, Professor	Committee	
Shuyang Zhang	Female	1963.3	Department of cardiology in Peking Union Medical College Hospital, Professor	Committee	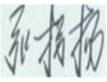
Hua Bai	Female	1970.2	Department of cardiology in Peking Union Medical College Hospital, Physician	Committee	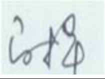

The ethics committee is independent and comply with ICH GCP, China GCP, local laws and regulations. All Committees present during their term of service. The ethics committee will keep confidential of the reviewed clinical research material, the results and relevant content discussed by the ethics committee meeting, and have no conflict of interest with the research project. If any serious adverse events occurred during the trial, please notify the ethics committee within 24 hours, and report the clinical trials progress in accordance with the review follow-up frequency. Drug clinical trials ethics committee of Peking Union Medical College Hospital address: Beijing Damucang Hutong No.41.

Contacts: Yan Sun. Phone number: 010-69158355. Hereby Declare.

Peking Union Medical College Hospital Ethics Committee

Attachment:

1. New drug certification
2. Drug testing report
3. Professional leading group approval report and principal investigator application report
4. Pharmaceutical GMP certificate and enterprise legal person business license
5. Drug instruction
6. Clinical trial protocol
7. Informed consent
8. CRF
9. Principal investigator, principal participant's resume and copy of GCP training certification
10. Researcher’s meeting minutes

Peking Union Medical College Hospital Ethics Committee
